# A Feed-Forward Circuit Linking Wingless, Fat-Dachsous Signaling, and the Warts-Hippo Pathway to *Drosophila* Wing Growth

**DOI:** 10.1371/journal.pbio.1000386

**Published:** 2010-06-01

**Authors:** Myriam Zecca, Gary Struhl

**Affiliations:** 1 Howard Hughes Medical Institute, Columbia University College of Physicians and Surgeons, New York, New York, United States of America; 2 Department of Genetics and Development, Columbia University College of Physicians and Surgeons, New York, New York, United States of America; Biozentrum der Universitaet Basel, Switzerland

## Abstract

During development, the *Drosophila* wing primordium undergoes a dramatic increase in cell number and mass under the control of the long-range morphogens Wingless (Wg, a Wnt) and Decapentaplegic (Dpp, a BMP). This process depends in part on the capacity of wing cells to recruit neighboring, non-wing cells into the wing primordium. Wing cells are defined by activity of the selector gene *vestigial* (*vg*) and recruitment entails the production of a *vg*-dependent “feed-forward signal” that acts together with morphogen to induce *vg* expression in neighboring non-wing cells. Here, we identify the protocadherins Fat (Ft) and Dachsous (Ds), the Warts-Hippo tumor suppressor pathway, and the transcriptional co-activator Yorkie (Yki, a YES associated protein, or YAP) as components of the feed-forward signaling mechanism, and we show how this mechanism promotes wing growth in response to Wg. We find that *vg* generates the feed-forward signal by creating a steep differential in Ft-Ds signaling between wing and non-wing cells. This differential down-regulates Warts-Hippo pathway activity in non-wing cells, leading to a burst of Yki activity and the induction of *vg* in response to Wg. We posit that Wg propels wing growth at least in part by fueling a wave front of Ft-Ds signaling that propagates *vg* expression from one cell to the next.

## Abstract 2

The secreted morphogen Wingless promotes *Drosophila* wing growth by fueling a wave front of Fat-Dachsous signaling that recruits new cells into the wing primordium.

## Author Summary

Under normal conditions, animals and their various body parts grow until they achieve a genetically predetermined size and shape—a process governed by secreted organizer proteins called morphogens. How morphogens control growth remains unknown. In *Drosophila*, wings develop at the larval stage from wing primordia. Recently, we discovered that the morphogen Wingless promotes growth of the *Drosophila* wing by inducing the recruitment of neighboring cells into the wing primordium. Wing cells are defined by the expression of the “selector” gene *vestigial*. Recruitment depends on the capacity of wing cells to send a short-range, feed-forward signal that allows Wingless to activate *vestigial* in adjacent non-wing cells. Here, we identify the molecular components and circuitry of the recruitment process. We define the protocadherins Fat and Dachsous as a bidirectional ligand-receptor system that is controlled by *vestigial* to generate the feed-forward signal. Further, we show that the signal is transduced by the conserved Warts-Hippo tumor suppressor pathway via activation of its transcriptional effector Yorkie. Finally, we propose that Wingless propels wing growth by fueling a wave front of Fat-Dachsous signaling and Yorkie activity that propagates *vestigial* expression from one cell to the next.

## Introduction

Growth is a fundamental property of animal development. Under normal conditions, animals of a given species, as well as their various body parts, achieve a characteristic size, shape, and pattern under tight genetic control. However, the basis of this control is poorly understood.

Morphogens, such as secreted factors of the Wingless/Int (Wnt), Bone Morphogenetic Protein (BMP), and Hedgehog (Hh) families, control growth. For example, in the classic paradigm of the *Drosophila* wing, the morphogens Wingless (Wg, a Wnt) and Decapentaplegic (Dpp, a BMP) drive a rapid ∼200-fold increase in cell number and mass that occurs during larval life [1],[2],[3],[4],[5]. Removal of either morphogen results in truncated wings [4],[5],[6],[7]. Conversely, their ectopic expression induces supernumerary wings [1],[2],[4],[5],[8].

Another system involved in growth is the evolutionarily conserved Warts-Hippo tumor suppressor pathway [9],[10],[11],[12]. This pathway includes the Warts (Wts) and Hippo (Hpo) kinases, the FERM domain proteins Expanded (Ex) and Merlin (Mer), and the accessory proteins Salvador (Sav) and Mob-as-tumor-suppressor (Mats). All of these proteins limit growth by mediating the phosphorylation and cytosolic retention of the transcriptional co-activator Yorkie (Yki)/YES Associated Protein (YAP) [9],[11], preventing Yki from up-regulating genes that promote growth [9],[13],[14].

In *Drosophila*, two protocadherins, Dachsous (Ds) and Fat (Ft), have been implicated as a ligand-receptor pair that acts, via the atypical myosin Dachs (D), to regulate Wts kinase activity [11],[15],[16],[17],[18],[19]. Previous studies have shown that morphogens such as Wg, Dpp, and Hh direct the formation of opposing, tissue-wide gradients of Ds and Ft activity [20],[21],[22],[23],[24]. Further, it has been proposed that the differential (i.e., slope) of Ds-Ft signaling across each cell sets the level of Wts activity and thereby governs the rate of growth and division on a cell-by-cell basis [24],[25] (see also [26]). In support, experiments that create sharp disparities in morphogen receptor activity or Ds-Ft signaling down-regulate Wts-Hpo activity and induce abnormal growth [24],[25],[27]. Conversely, experiments that flatten Ds-Ft signaling (e.g. uniform over-expression of Ds) suppress growth [22],[24],[25],[28].

Ft and Ds are also important for planar cell polarity (PCP), in which cells within epithelial sheets adopt a common orientation, e.g. as manifest by their secreting hairs that point in the same direction [20],[21],[29],[30],[31]. In this case, the ligand-receptor relationship between the two proteins appears more complex [23],[32]. Cells that express only Ds or only Ft can polarize their neighbors, whereas cells that lack either Ds or Ft cannot respond to their neighbors. Hence, in PCP, Ds and Ft each have intrinsic signaling activities, and both are required to receive and transduce each signal [23],[32].

Recently, we defined a new mechanism for the control of *Drosophila* wing growth by morphogen [33],[34]. Focusing on Wg, we showed that morphogen propels growth at least in part by fueling a reiterative process of recruitment of non-wing cells into the wing primordium. Recruitment depends on a special, auto-regulatory property of *vestigial* (*vg*), the selector gene that defines the wing state [35]. This is the capacity of *vg* expressing cells to send a feed-forward (FF) signal that induces neighboring cells to activate *vg* in response to Wg [33],[34]. Early in larval life, specialized “border” cells along the boundary between the dorsal (D) and ventral (V) compartments are induced to express Vg and secrete Wg. These cells initiate the FF recruitment process, which then reiterates, propagating *vg* expression from cell to cell in response to Wg spreading from the border cells.

In our initial analysis of the recruitment process, we speculated that Ft and Ds might be involved in the FF mechanism [33]. Here, we confirm this speculation and show that Ft is required for cells both to send and, together with Ds, to receive the FF signal, concordant with the dual ligand and receptor activities of both proteins in PCP. Further, we show that Ft and Ds transduce the FF signal via D, the Wts-Hpo pathway, and Yki to activate *vg* expression and initiate a new cycle of FF signaling. Based on these findings, we posit that Wg (and likely Dpp) promote wing growth by fueling the propagation of a wave front of Ft-Ds signaling that transiently suppresses the Wts-Hpo pathway and elevates Yki activity to recruit new cells into the wing primordium.

## Results

### The vg FF Signal

**Figure 1:**
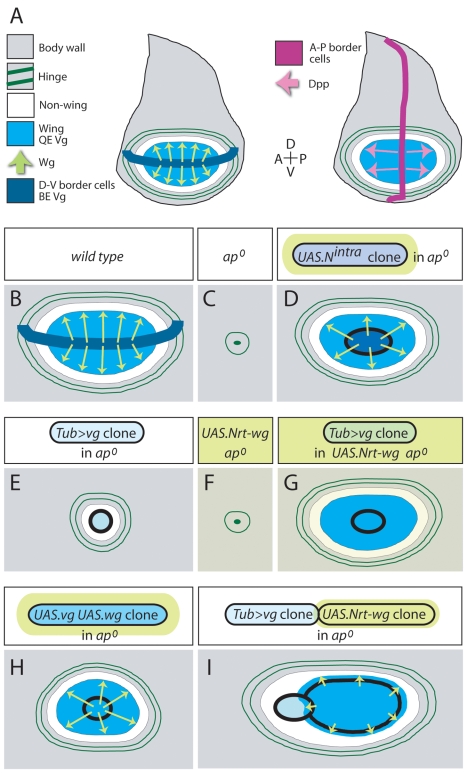
Feed-forward signaling: context and criteria. (A) Context. Two diagrams of the mature wing imaginal disc are shown, depicting control of wing growth by Wg (left) and Dpp (right) and keys for the relevant primordia, signals, and gene expression domains. Early in larval life, the wing disc is subdivided into distal (prospective wing; turquoise/white) and proximal (prospective hinge and body wall; grey) domains. Feed-forward (FF) signaling operates only in the distal domain, to induce non-wing cells (white) to enter the wing primordium (turquoise). Both domains are further subdivided into D and V compartments by activity of the selector gene *ap* in the D compartment (not depicted). DSL-Notch signaling across the D-V compartment boundary defines a population of specialized border cells (dark blue) that express *wg* and *vg*, the latter mediated by the *vg* Boundary enhancer (BE). The wing disc is also divided into anterior (A) and posterior (P) compartments, with A cells just anterior to the A-P boundary secreting Dpp (for simplicity only shown in A). Following the D-V segregation, *vg* expressing wing cells send a short-range feed-forward (FF) signal (not depicted) that acts together with Wg and Dpp to activate Quadrant enhancer (QE) dependent *vg* expression (turquoise) in abutting non-wing cells; newly recruited wing cells serve as a source for new FF signal, propagating recruitment of neighboring non-wing cells into the wing primordium in response to Wg and Dpp (see Figure 8A). Wg and Dpp are also required (i) to maintain QE-dependent *vg* expression in cells once they are recruited into the wing primordium, (ii) to sustain the survival and growth of wing cells, so defined, and (iii) to act indirectly, through the action of Vg, to produce an additional signal that induces proliferation of surrounding non-wing cells for recruitment into the growing wing primordium [33],[34]. The hinge primordium, which encircles the prospective wing, contains two concentric rings of *wg* expressing cells (dark green) that serve as landmarks as well as potential sources for cryptic Wg signal in *ap^o^* discs. (B–I) Criteria. FF signaling is monitored by assaying QE-dependent gene expression. (B) wild type. Here, as in the remaining panels, the genotype is indicated above and the QE response below for each of several experimental paradigms used to define the FF signal [33],[34]. Wg signal is depicted by Chartreuse arrows or wash. QE activity and formation of wing tissue (turquoise) indicates a positive response. (C) The *ap^o^* condition serves as the ground state for assaying FF signaling. In the absence of *ap*, no D-V segregation occurs, no D-V border cells are specified and the nascent wing primordium ceases to express *vg*, yielding a population of “non-wing” cells that either die or sort out during subsequent development, unless they are induced to activate QE-dependent *vg* expression in response to Wg and the FF signal generated by an experimental manipulation (Dpp is provided, independently, by A-P border cells). As diagrammed, mature *ap^o^* discs lack wing (turquoise) and non-wing (white) territories, as well as the distal portion of the hinge primordium, reducing the inner ring of Wg expression to a small patch, encircled by a rudimentary outer ring. (D) Cells that express constitutively active forms of Notch in *ap^o^* discs (e.g., *UAS.N^intra^* clones) behave like ectopic D-V border cells. They express *wg* and *vg*, induce neighboring non-wing cells to activate QE-dependent *vg* expression, and recruit surrounding cells to join a rapidly expanding wing primordium. (E,F) Providing only Vg expressing cells [e.g., *Tub>vg* clones; (E)] or only ectopic Wg signal [uniform expression of Neurotactin-Wg (*UAS.Nrt-Wg*), a membrane tethered form of Wg] fails to induce QE activity, except within *Tub>vg* expressing cells, where the combination of cryptic Wg input and exogenous Vg activity weakly activates the QE cell-autonomously (E, light turquoise wash). (G–I) Generating Vg expressing cells in the presence of Wg signal, whether in the form of ubiquitous Nrt-Wg expression (G), co-expression of ectopic Wg (H), or abutting clones of Nrt-Wg expressing cells (I), induces long-range propagation of QE-dependent *vg* expression and rescue of wing tissue. Note that in the last condition (I), FF signaling can propagate throughout the Nrt-Wg clone and extend to abutting wild type cells (which receive the Nrt-Wg signal) but does not go further owing to inadequate Wg signal in the surround.

The main phase of wing growth begins early in larval life with the segregation of the prospective wing primordium into D and V compartments [36],[37],[38]. Short-range Notch signaling across the D-V boundary activates the *vg* Boundary Enhancer (BE) to generate a stripe of *vg* expressing “border cells” [35],[39]. It also induces border cells to secrete Wg [40],[41],[42], which activates and sustains *vg* expression in surrounding cells via the *vg* Quadrant Enhancer (QE) (Figure 1A, 1B) [4],[5],[33],[34],[35], driving the rapid increase of the wing primordium from a population of ∼25–50 cells to one of ∼5,000–10,000 cells.

**Figure 2:**
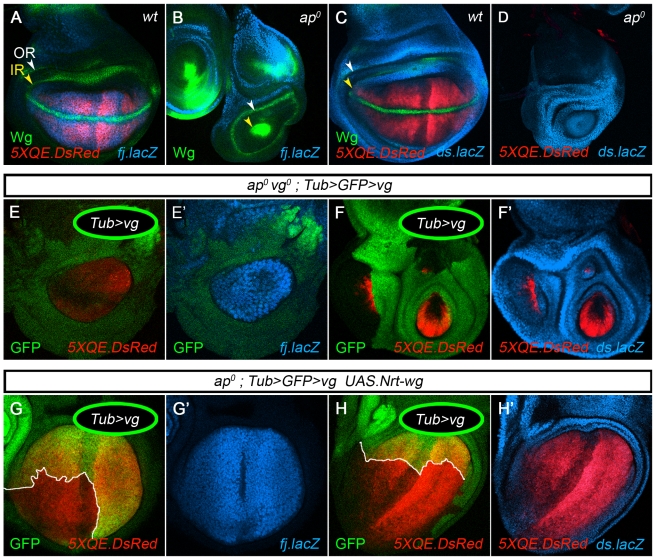
Vestigial activates *four-jointed* and represses *dachsous*. (A–D) *fj-lacZ*, *ds-lacZ*, and *5XQE.DsRed* reporter expression in mature wild type and *ap^o^* discs counter-stained for Wg (A–C, only); note that *5XQE.DsRed* expression is reduced in the vicinity of the A-P compartment boundary, as also apparent in (G,H). In wild type discs (A,C), *fj-lacZ* and *5XQE.DsRed* are co-expressed in the wing pouch in a domain complementary to that of *ds-lacZ* (the inner (IR) and outer (OR) rings of Wg in the hinge primordium are indicated by yellow and white arrow heads). In *ap^o^* discs (B,D), the wing pouch is absent, as indicated by the collapse of the IR to a small circular patch surrounded by the OR: *ds-lacZ* is expressed uniformly in place of *fj-lacZ* and *5XQE.DsRed* in the territory encircled by the OR. (E,F). *fj-lacZ*, *ds-lacZ*, and *5XQE.DsRed* reporter expression in *ap^o^ vg^o^* discs that contain clones of *Tub>vg* cells (marked by the absence of GFP). Clones located in the prospective wing domain develop cell-autonomously as wing tissue and express *fj-lacZ* and *5XQE.DsRed* instead of *ds-lacZ*. (G,H) Clones of *Tub>vg* cells in *UAS.Nrt-wg* expressing *ap^o^* discs (as in Figure 1G). Clones (outlined in white, marked by the absence of GFP) induce the long-range propagation of QE-dependent *vg* expression, as visualized by the domain of *5XQE.DsRed* expression. Recruitment into wing tissue correlates with the up-regulation of *fj-lacZ* expression and the down-regulation of *ds-lacZ* expression. Here, and in subsequent figures, genotypes, clone markers, and antibody stains are indicated on the panels, coded by color (clones marked by the absence of GFP are shown as open circles with green borders; those marked positively are shown as filled circles), or in boxes above the panels (in all cases in which a *UAS* transgene is indicated in a box, its expression is driven by a Gal4 driver that is uniformly active in the prospective wing territory; white/turquoise domain as in Figure 1A, 1B; see Materials and Methods for exact genotypes).

D-V compartmentalization depends on the heritable activation of the selector gene *apterous* (*ap*) in D, but not V, cells [36],[43]. In *ap* null discs (henceforth *ap^o^* discs), the D-V segregation fails, *vg* and *wg* expressing border cells are not specified, and the nascent wing primordium is subsequently lost (Figures 1C, 2B). However, it is possible to rescue wing development in *ap^o^* discs by experimental protocols that provide both Wg and a population of ectopic Vg expressing cells (Figure 1D–I; Figure 2G,H) [33],[34]. Under these conditions, the ectopic Vg expressing cells induce neighboring cells that receive Wg to activate QE-dependent *vg* expression (turquoise shading in Figure 1), and these newly recruited *vg* expressing cells can similarly induce their non-expressing neighbors, the process reiterating to increase the size of the wing primordium [33],[34].

These results establish that Vg expressing cells send a short-range, inductive signal that is required, together with Wg, to activate QE-dependent *vg* expression in neighboring cells. We term this Vg-dependent, Vg-inducing signal the FF signal [33],[34].

In the experiments below, we exploit the same experimental protocols (Figure 1C–I) to identify gene functions that are required to send and/or to receive the FF signal. We monitor the results of these manipulations by assaying QE activity as visualized by the expression of *1XQE.lacZ* and *5XQE.DsRed* reporters, as well as endogenous Vg [33],[35]; all three responses behave similarly, and we use them interchangeably.

### FF Signaling Correlates with Steep, Vg-Dependent Differentials in Opposing Ft and Ds Signals

During normal development, *vg* activity drives production of the FF signal, and transduction of the signal occurs at the periphery of the wing primordium, where recruitment occurs. Strikingly, two genes involved in Ft-Ds signaling, *four-jointed* (*fj*) and *ds*, itself, are expressed at peak levels in complementary domains that abut at the wing periphery, *fj* in the *vg* ^ON^ domain (Figure 2A) and *ds* in the *vg* ^OFF^ surround (Figure 2C). *fj* encodes a Golgi resident ecto-kinase that functions in PCP to potentiate signaling by Ft and inhibit signaling by Ds [20],[21],[23],[44],[45],[46]. Hence, *vg* may generate the FF signal by activating *fj* transcription and repressing *ds* transcription to create steep and opposing differentials in Ft and Ds signaling between wing and non-wing cells.

One prediction of this hypothesis is that Vg should be both necessary and sufficient to activate *fj* and repress *ds* in prospective wing cells. To test this, we used *fj-lacZ* and *ds-lacZ* reporters to monitor the consequences of ectopically expressing Vg in *ap^o^* discs.

Mature *ap^o^* discs lack the wing primordium as well as adjacent portions of the hinge primordium (Figures 1C, 2B); the remaining cells (which correspond to the rest of the prospective hinge and body wall) express high levels of *ds-lacZ* (Figure 2D) but not *fj-lacZ* (Figure 2B). To determine if Vg is sufficient to activate *fj-lacZ* and repress *ds-lacZ*, we generated clones of *Tub>vg* cells in *ap^o^* discs that are also *vg^o^* (to eliminate any contribution from endogenous Vg activity). Such clones express moderate levels of exogenous Vg, a few fold lower than the peak endogenous level observed in wild type discs, and rescue wing development cell-autonomously [33]. They also express *fj-lacZ* and repress *ds-lacZ* (Figure 2E, 2F). Thus, ectopic Vg acts cell-autonomously to up-regulate *fj* and down-regulate *ds* in *ap^o^ vg^o^* discs.

A second prediction of the hypothesis that *vg* generates the FF signal by activating *fj* and repressing *ds* is that FF propagation should correlate with the up-regulation of *fj* transcription at the expense of *ds* transcription. To test this we analyzed the effects of *Tub>vg* clones on *fj-lacZ* and *ds-lacZ* expression in *ap^o^* discs supplemented with exogenous Wg, a context in which they induce long-range propagation of QE-dependent *vg* expression and wing growth (as in Figure 1G; [33]).

As previously shown, *Tub>vg* clones generated in such discs cell-autonomously activate peak levels of QE-dependent *vg* expression and induce the long-range propagation of QE-dependent *vg* expression in surrounding tissue (Figure 2G, 2H; [33]). They also induce the long-range propagation of *fj-lacZ* expression at the expense of *ds-lacZ* expression (Figure 2G, 2H), establishing a correlation between FF propagation and the control of *fj* and *ds* transcription by *vg*.

Two additional properties of *Tub>vg* clones are important to note. First, *Tub>vg* clones activate *fj-lacZ* and repress *ds-lacZ* only in the prospective wing (white/turquoise territory depicted in Figure 1A, 1B) and not in the prospective hinge and body wall (grey territory in Figure 1A, 1B), as is also the case for activation of the QE (Figure 2E, 2F). This is expected, as the FF recruitment process operates only in the prospective wing, where the selector gene *teashirt* is off, and not in the more proximal domains where it is on [33],[34].

Second, *Tub>vg* clones activate QE-dependent *vg* expression, albeit weakly, in *ap^o^* discs, even in the absence of exogenous Wg, despite the fact that these discs are devoid of D-V border cells, the normal source of Wg required for QE activity. As previously shown [33],[34], this response depends on low levels of cryptic Wg, possibly emanating from the surrounding hinge primordium, which allows the QE to be activated cell-autonomously by the exogenous Vg produced by the *Tub>vg* transgene.

Both the presence of cryptic Wg signal in *ap^o^* discs as well as the restriction of FF propagation to the prospective wing territory are relevant preconditions for the experiments presented below.

### Ft and Ds Suppress QE-Dependent *vg* Expression in the Absence of FF Signal

Given that *ft^o^* and *ds^o^* discs show extra wing growth, we previously speculated that Ft and Ds normally suppress QE activity in non-wing cells and that the FF signal acts as an antagonist to alleviate this suppression, allowing the QE to respond to Wg [33]. Accordingly, the removal of either protein should mimic receipt of the FF signal and alleviate the block to Wg-dependent activation of the QE. We tested this prediction by assaying QE activity in *ft^o^ ap^o^* and *ds^o^ ap^o^* discs, either in the presence or absence of exogenous Wg.

**Figure 3:**
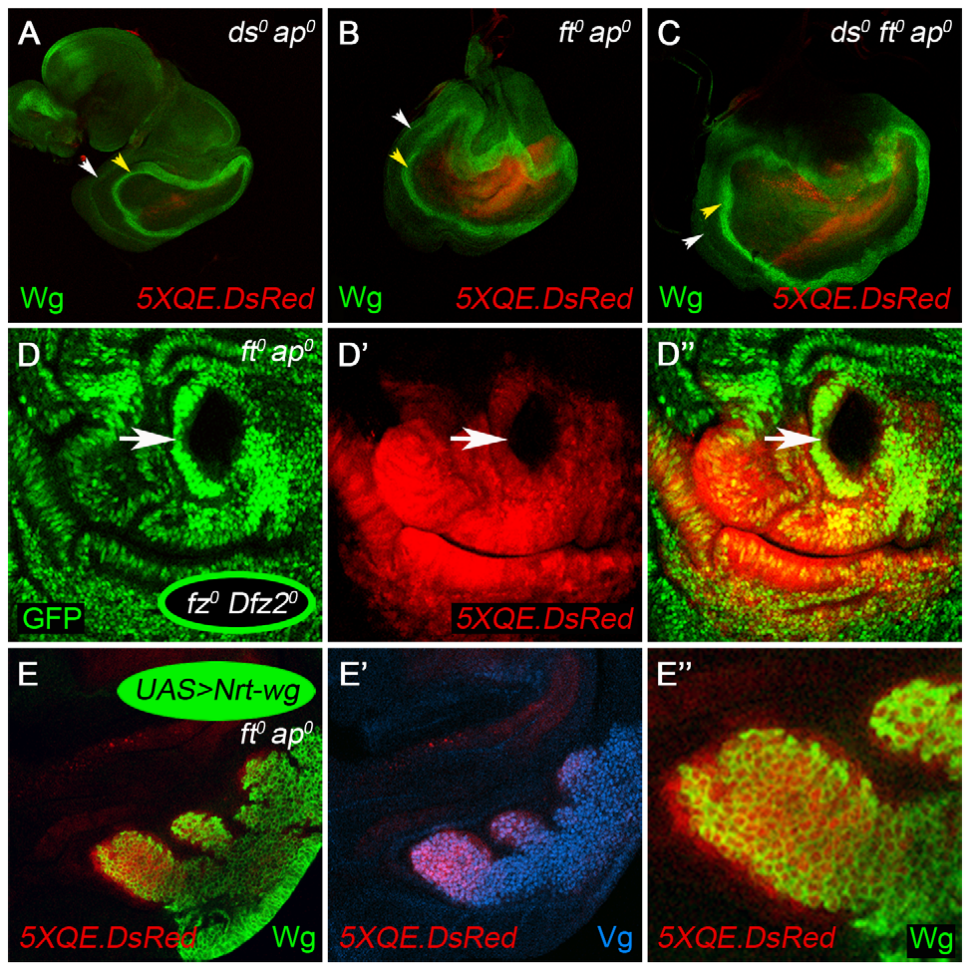
Fat and Dachsous are required to block Quadrant enhancer activity in the absence of feed-forward signal. (A–C) Removal of either, or both, Ft and Ds causes constitutive, low-level QE activity (monitored by *5XQE.DsRed* expression) in *ap^o^* discs. *ap^o^* discs that are *ds^o^*, *ft^o^*, or *ds^o^ ft^o^* form wing pouches that express the *5XQE.DsRed* reporter and are encircled by the Wg IR and OR, in contrast to single mutant *ap^o^* discs (Figure 2B). Note that the level of *5XQE.DsRed* expression is very low, especially in the *ds^o^ ap^o^* disc, consistent with the presence of only cryptic levels of Wg; note also that some DsRed expression within the rescued pouch appears outside of the Wg IR because it is in a fold, underneath. (D) The *5XQE.DsRed* response observed in *ft^o^ ap^o^* discs depends on Wg input. *5XQE.DsRed* expression is lost in clones of *fz^o^ Dfz2^o^* cells in the wing pouch of *ft^o^ ap^o^* discs (a single *fz^o^ Dfz2^o^* clone is indicated by an arrow). (E) Clones of *UAS.Nrt-wg* cells induce normal, peak expression of both the *5XQE.DsRed* reporter and endogenous Vg within the clone and in adjacent cells (the low levels of *5XQE.DsRed* and Vg expression in surrounding cells can only be detected, as in A–D, using more intense laser illumination).

As described above, *ap^o^* discs do not activate QE-dependent *vg* expression and fail to sustain a wing primordium (Figures 1C and 2B) [33],[34]. In contrast, *ft^o^ ap^o^* discs show at least partial rescue of the wing primordium, and cells within the primordium express both *5XQE.DsRed* and Vg, albeit at barely detectable levels (Figure 3B and unpublished data; the rescue observed is due to this low level Vg activity, as it does not occur in *ft^o^ ap^o^ vg^o^* discs). Hence, prospective wing cells in these discs behave as if they have constitutively activated the FF signal transduction pathway but can mount only a weak QE response owing to the low levels of cryptic Wg available [34].

This interpretation is supported by two experiments that show that QE activity in *ft^o^ ap^o^* discs is Wg dependent. First, the QE response is abolished in clones of *fz^o^ Dfz2^o^* cells, which are unable to transduce Wg (Figure 3D) [47]. Second, clones of cells that express a membrane tethered form of Wg (Nrt-Wg; [4],[5]) under Gal4/UAS control (henceforth, *UAS.Nrt-wg* clones) drive peak levels of Vg and *5XQE.DsRed* expression in *ft^o^ ap^o^* discs, both within the clones and in abutting cells (Figure 3E; unpublished data). By contrast, Nrt-Wg fails to rescue Vg expression or wing development in *ap^o^* discs that are wild type for *ft* (Figure 1F) [33], confirming that it is the absence of Ft activity in *ft^o^ ap^o^* discs that allows them to activate the QE in response to Wg.

*ds^o^ ap^o^* discs behave similarly to *ft^o^ ap^o^* discs, except that they express even lower levels of *5XQE.DsRed* and Vg, and the rescued wing primordium is smaller (Figure 3A; unpublished data). Nevertheless, as in *ft^o^ ap^o^* discs, both responses are activated to peak levels by *UAS.Nrt-wg* clones (Figure S1). The effect of removing *ds* appears to be additive to that of removing *ft*: the rescued wing primordium in triply mutant, *ds^o^ ft^o^ ap^o^* discs tend to be larger, on average, than those in *ft^o^ ap^o^* discs (Figure 3B, 3C). The distinct and additive effects of removing Ft and Ds suggest that neither condition corresponds to normal, peak activation of the FF transduction pathway. Instead, as we describe below, each appears to lock the FF transduction pathway into a state of weak, constitutive activity, rendering the level of QE activity refractory to the presence or absence of incoming FF signal.

We conclude that Ft and Ds are normally required in non-wing cells to block QE activity and that receipt of the FF signal alleviates this suppression, allowing the QE to be activated by Wg. Below, we present evidence that Ft, itself, corresponds to the FF signal sent by wing cells and that Ft and Ds function in non-wing cells to receive and transduce this signal.

### Ft Is Required for Sending the FF Signal

If, as we posit above, *vg* generates the FF signal by up-regulating Ft signaling at the expense of Ds signaling, wing cells should require *ft*, but not *ds*, to induce QE-dependent *vg* expression in neighboring non-wing cells. To test this, we generated *ds^o^* and *ft^o^* clones in *ap^o^* discs. Given that the loss of either Ds or Ft mimics reception of the FF signal, such clones should cell-autonomously activate QE-dependent *vg* expression and survive as wing tissue in *ap^o^* discs. Accordingly, they should serve as ectopic sources of FF signal, allowing us to determine if their capacity to send FF signal depends on either Ds or Ft activity.

**Figure 4:**
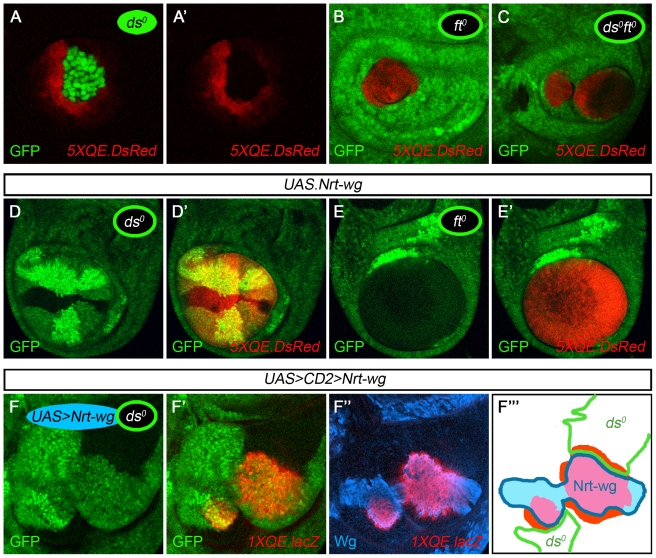
Fat is required in *vestigial* expressing cells to send feed-forward signal. (A–C) Clones of *ds^o^*, *ft^o^*, and *ds^o^ ft^o^* cells in *ap^o^* discs. The *ds^o^* clone (A) is marked positively by the expression of GFP to allow the non-autonomous induction of *5XQE.DsRed* expression to be clearly distinguished from the clone. Conversely, *ft^o^* and *ds^o^ ft^o^* clones (B,C) are marked negatively, by the absence of GFP, to visualize the strictly cell-autonomous expression of the *5XQE.DsRed* transgene. *5XQE.DsRed* is expressed only at cryptic low levels within *ds^o^*, *ft^o^*, and *ds^o^ ft^o^* clones (as in entirely *ds^o^ ap^o^, ft^o^ ap^o^*, and *ds^o^ ft^o^ ap^o^* discs; Figure 3A–C) and is not detectable in (A) at the level of laser illumination used to generate this image. However, *ds^o^* clones induce surrounding, wild type cells to express much higher levels of *5XQE.DsRed* expression, in contrast to *ft^o^* and *ds^o^ ft^o^* clones, indicating that they generate ectopic FF signal. We infer that the absence of Ds activity in the *ds^o^* cells constitutively activates the FF signal transduction pathway but only at a low level relative to the peak response of surrounding, wild type cells to ectopic FF signal sent by the clone. (D,E) Clones of *ds^o^* and *ft^o^* cells in *ap^o^* discs that express *UAS.Nrt.wg* uniformly under Gal4 control (“*UAS.Nrt-wg*” discs in all subsequent panels; the non-autonomous induction of *5XQE.DsRed* expression appears as yellow in D'). *ds^o^* clones activate *5XQE.DsRed* expression cell-autonomously and serve as a potent source of FF signal, inducing surrounding cells to express the *5XQE.DsRed* reporter and join a growing wing primordium. Conversely, most *ft^o^* clones show only a strictly cell-autonomous response (exceptions appear to be associated with ectopic FF signal generated by sibling *ft^+^/ft^+^* clones, as documented in Figure S2). (F) An *ap^o^* disc containing abutting *ds^o^* and *UAS.Nrt-wg* clones marked, respectively, by the absence of GFP and the expression of Nrt-Wg (F''' depicts the experiment in cartoon form). The *ds^o^* clones behave like *Tub>vg* clones (Figure 1I): they induce high levels of QE-dependent *vg* expression (monitored by both *1XQE.lacZ* and endogenous Vg expression) in the abutting Nrt-Wg cells within the prospective wing domain. Moreover, QE activation propagates over many cell diameters within the Nrt-Wg clone and extends to adjacent cells across the clone border. Finally, the QE response is also up-regulated in *ds^o^* cells that abut the Nrt-Wg clone, in response to the tethered Wg signal.

As expected from the behavior of entirely mutant *ds^o^ ap^o^* and *ft^o^ ap^o^* discs (Figure 3A, 3B), both *ds^o^* and *ft^o^* clones survive and develop as wing tissue in *ap^o^* discs (Figure 4A, 4B). However, they express only cryptic, low levels of *5XQE.DsRed* and Vg (Figure 4B; unpublished data; see also Figure 4D, 4E), like cells within the wing primordia of *ds^o^ ap^o^* and *ft^o^ ap^o^* mutant discs (Figure 3A, 3B). Strikingly, *ds^o^* clones also act non-autonomously to induce higher levels of QE activity in neighboring cells (Figure 4A). In contrast, *ft^o^* clones do not (Figure 4B). Thus, it appears that Ft, but not Ds, is required to send the FF signal.

To determine if the non-autonomous induction of QE activity by *ds^o^* clones is due specifically to Ft activity in the mutant cells, we generated *ds^o^ ft^o^* clones. Such clones behave like *ft^o^* clones in showing strictly cell-autonomous QE activity (Figure 4C). Hence, *ds^o^* cells require Ft to generate ectopic FF signal.

Assaying FF signaling is limited in *ap^o^* discs by the dependence of QE activity on cryptic Wg input (Figure 3D, 3E; [34]). We therefore repeated the *ds^o^* and *ft^o^* clone experiments, this time supplementing this cryptic Wg signal with uniformly expressed Nrt-Wg (as in Figure 1G).

In the presence of Nrt-Wg, *ds^o^* clones expressed peak levels of Vg and *5XQE.DsRed* cell-autonomously and induced the long-range propagation of both responses in surrounding cells (Figure 4D; unpublished data). Similar results were obtained when we supplied exogenous Wg by generating *ds^o^* clones that express a *UAS.wg* transgene (using the MARCM technique [48]; unpublished data) and by generating *UAS.Nrt-wg* expressing clones next to *ds^o^* clones in the same disc (Figure 4F). In the latter case, the *ds^o^* clones behave indistinguishably from *Tub>vg* clones in the original experimental paradigm used to define the FF signal (Figure 1I; [33]): they induce the long-range propagation of peak levels of Vg and *5XQE.DsRed* expression in abutting *UAS.Nrt-wg* clones (an effect that can extend to the immediate, wild type neighbors of the *UAS.Nrt-wg* clone). These results confirm that *ds^o^* clones serve as ectopic sources of FF signal, capable of inducing QE-dependent *vg* expression in neighboring cells, provided that the responding cells also receive Wg.

In contrast, and with only limited exceptions (Figure S2), *ft^o^* clones elicited a strictly cell autonomous response, both in Nrt-Wg expressing *ap^o^* discs (Figure 4E) and when exogenous Wg was supplied using the MARCM technique (unpublished data). Such *ft^o^* clones form ectopic wing primordia composed solely of mutant cells, excluding even cells of their wild type sibling clones from contributing to the rescued wing tissue (Figure 4E; the sibling clone is marked by elevated GFP staining; compare with the inclusion of the corresponding sibling cells in the case of *ds^o^* clones, Figure 4D). The cell autonomous response of these *ft^o^* clones is especially significant because all cells within such clones express peak levels of Vg and *fj-lacZ* (unpublished data) and hence should be potent sources of FF signal; nevertheless they behave as if devoid of the capacity to signal. Note that this failure cannot be attributed to a generic inability of *ft^o^* cells to send intercellular signals. First, *ft^o^* clones repolarize their neighbors, whereas *ds^o^ ft^o^* clones do not, indicating that they have the capacity to send the Ds PCP signal [21],[23],[30],[44]. Second, we have verified by experiment that *ft^o^* clones in the wing primordium can also send DSL-Notch, Wg, and Dpp signals (Figure S3).

Thus, we conclude that Ft is normally required in *vg* expressing cells to send the FF signal.

### Ft and Ds Are Required for Receiving the FF Signal

**Figure 5:**
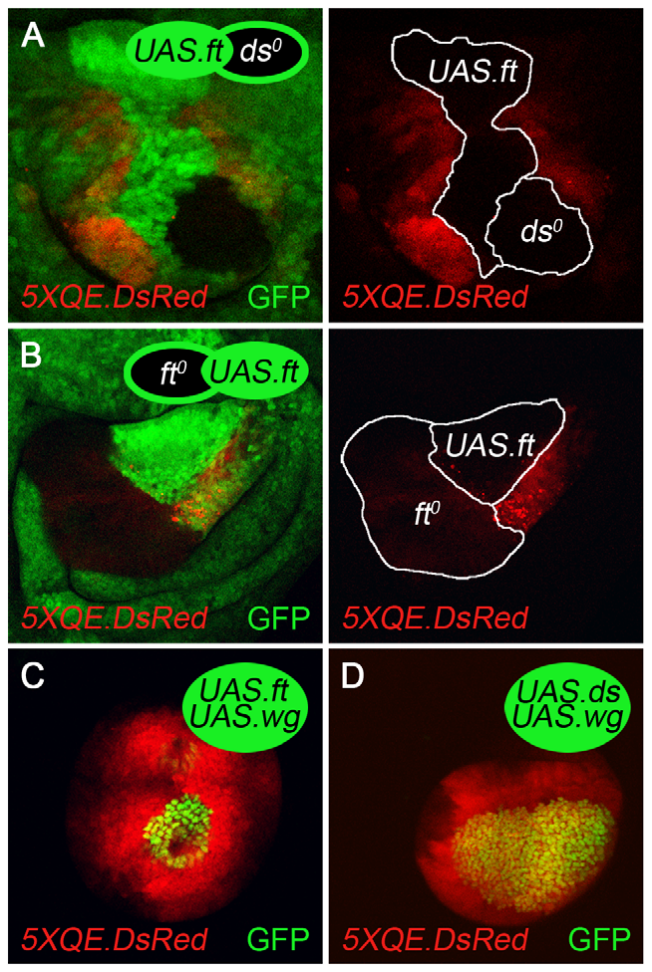
Generation and transduction of feed-forward signal by Fat and Dachsous in *ap^o^* discs. (A) Abutting, sibling clones of *UAS.ft* and *ds^o^* cells marked, respectively, by high (2×) or no (0×) GFP expression in a background of moderate (1×) GFP expressing cells, and outlined in white. The *UAS.ft* clone has induced *5XQE.DsRed* expression in neighboring wild type cells but not in the abutting *ds^o^* cells. As noted in the legend to Figure 4A, the loss of *ds* is associated with the cell-autonomous activity of the *5XQE-DsRed* reporter but only at cryptic, low level relative to the response induced in wild type cells by receipt of FF signal (and hence not detected at the level of laser illumination used in this image). (B) Abutting, sibling clones of *UAS.ft* and *ft^o^* cells (marked as in A). The result is the same: like *ds^o^* cells, the *ft^o^* cells are refractory to induction of the *5XQE-DsRed* transgene by abutting *UAS.ft* cells, in contrast to neighboring wild type cells. Similarly, as in the case of *ds^o^* clones, *5XQE.DsRed* transgene is expressed constitutively, but only at cryptic low level, in *ft^o^* clones (as in Figure 4B) and is not readily detectable in this image. (C,D) *UAS.ft UAS.wg* (C) and *UAS.ds UAS.wg* (D) clones: The *5XQE.DsRed* transgene is strongly expressed both within, and in a halo around, each clone.

Ft and Ds have a complex ligand-receptor relationship in PCP: both proteins have intrinsic signaling activity, and both are required, together, to receive and transduce each of the signals [23]. Hence, as in PCP, Ft may be required both to generate the FF signal in wing cells and, together with Ds, to receive the FF signal in non-wing cells. To test this, we generated abutting, sibling clones (“twin spots”) in which one clone is *UAS.ft* and the other is either *ds^o^* or *ft^o^* and assayed for the capacity of the *UAS.ft clones* to induce QE activity in neighboring wild type, *ds^o^*, or *ft^o^* cells (Figure 5A, 5B).

*UAS.ft* clones express levels of Ft that are several fold higher than endogenous Ft (unpublished data) and generate ectopic FF signal in *ap^o^* discs, as monitored by the induction of *5XQE.DsRed* expression in adjacent wild type cells (Figure 5A, 5B; unpublished data). However, adjacent clones of *ft^o^* cells appear unresponsive to this FF signal, even when they abut the *UAS.ft* clones over an interface of many cell diameters (Figure 5B). Instead, they express *5XQE.DsRed* uniformly and at cryptic, low levels (as in Figure 3B), indicating that the FF transduction pathway is only weakly, albeit constitutively, active in *ft^o^* cells. Similarly, although clones of *ds^o^* cells can induce *5XQE.DsRed* expression in abutting wild type cells (as in Figure 4A), they too appear to be incapable of responding to adjacent *UAS.ft* clones (Figure 5A).

Thus, clonal over-expression of Ft is sufficient to generate an ectopic FF signal, but abutting *ds^o^* and *ft^o^* cells are refractory to this signal. Notably, we detect either no, or very little, expression of Vg or the *5XQE.DsRed* reporter in the Ft over-expressing cells, themselves. Hence, it appears that Ft itself, and not some other molecule under the control of Vg, is responsible for the FF signal sent by these cells.

Taken together with our preceding results, these findings indicate (i) that wing cells require Ft to generate FF signal and (ii) that non-wing cells require both Ft and Ds to receive the signal.

### Complementary Roles for Ft and Ds in FF Signaling

Although wing cells require Ft, but not Ds, to send the FF signal, cells undergoing recruitment are also in position to receive an opposing Ds signal coming from non-wing cells on the other side, raising the possibility that this Ds input may also contribute to activating the QE and recruiting cells into the wing primordium.

To assess this, we generated Ds over-expressing clones in *ap^o^* discs and asked if the resulting disparity in Ds signaling across the clone border is sufficient to induce the QE response in surrounding cells.

Clones of *UAS.ds* cells in *ap^o^* discs generate levels of Ds that are several fold higher than endogenous Ds (which is expressed at peak levels in these discs, owing to the absence of *vg* activity). In the absence of exogenous Wg, such *UAS.ds* clones had little effect on surrounding cells, only occasionally inducing *5XQE.DsRed* expression just outside the clone (unpublished data). However, when supplemented with exogenous Wg (using co-expression of a *UAS.wg* transgene), most *UAS.ds* clones induced *5XQE.DsRed* expression both within the clone and in surrounding cells (Figure 5D), as is also the case for *UAS.ft UAS.wg* clones (Figure 5C).

Thus, Ds over-expressing clones, like Ft over-expressing clones, can induce neighboring cells to activate QE-dependent *vg* expression in *ap^o^* discs, consistent with the possibility that recruitment of cells into the wing primordium normally depends on opposing Ft and Ds signals (Ft presented by wing cells and Ds presented by non-wing cells; see Discussion).

### Transduction of the FF Signal by the Wts-Hpo Pathway and Yki

The Wts-Hpo pathway is known to function downstream of Ft and Ds, as well as the atypical myosin D, in the generic control of growth by the transcriptional co-activator Yki [11],[15],[16],[17],[18],[19]. Hence, it may similarly link reception of the FF signal by Ft and Ds to the induction of QE-dependent *vg* expression. D activity normally promotes Yki activity by inhibiting the Wts kinase (which would otherwise phosphorylate Yki and prevent it from gaining access to the nucleus). Hence, if the FF signal is transduced by the Wts-Hpo pathway, manipulations that promote Yki action (e.g., removal of Ex or Wts, or over-expression of D or Yki [9],[11]) should activate QE-dependent Vg expression cell-autonomously, subject to Wg input. Moreover, such QE-Vg expressing cells should, themselves, act as sources of ectopic FF signal and induce surrounding cells to activate the QE. We tested these predictions by manipulating D, Ex, Wts, and Yki function in *ap^o^* discs, either with or without exogenous Wg.

*ap^o^* discs that uniformly over-express Yki, or which contain large clones of *wts^o^* cells, appear similar to *ft^o^ ap^o^* discs (Figure 3B), forming wing primordia that express *5XQE.DsRed* and Vg, albeit at barely detectable levels (Figure S1B, S1C; unpublished data). However, as in the case of *ft^o^ ap^o^* and *ds^o^ ap^o^* discs (Figure 3E; Figure S1A), clones of *UAS.Nrt-wg* cells in these *ap^o^ wts^o^* and *ap^o^ UAS.yki* discs induce peak levels of both Vg and *5XQE.DsRed* expression within the clone and in adjacent cells (Figure S1B, S1C), indicating that both the removal of Wts as well as the over-expression of Yki constitutively activate the FF signal transduction pathway.

**Figure 6:**
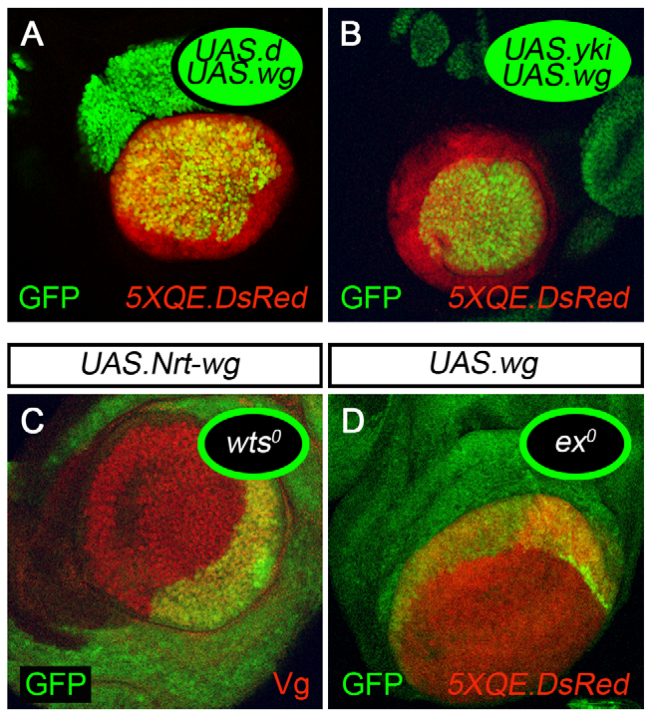
Reducing or bypassing Warts-Hippo activity ectopically activates Quadrant enhancer-dependent *vestigial* expression. (A,B) Clones of *UAS.d* (A) and *UAS.yki* (B) cells that co-express *UAS.wg* in *ap^o^* discs. Both clones activate QE dependent gene expression cell-autonomously as monitored by *5XQE.DsRed* expression. Both have also induced *5XQE.DsRed* expression in neighboring cells encircling the clone. (C,D) Clones of *wts^o^* (C) and *ex^o^* (D) cells in *UAS.Nrt-wg ap^o^* discs. Both clones activate QE-dependent gene expression cell-autonomously and have also induced QE activity in neighboring cells (monitored by Vg in C, and *5XQE.DsRed* expression in D).

Corroborating these results, clones of *UAS.d* and *UAS.yki* cells that co-express *UAS.wg* in *ap^o^* discs activate peak levels of *5XQE.DsRed* expression, cell-autonomously, and can also induce *5XQE.DsRed* expression in surrounding cells (Figure 6A, 6B). Likewise, clones of *ex^o^* or *wts^o^* cells generated in *UAS.Nrt-wg ap^o^* discs express peak levels of Vg and *5XQE.DsRed* cell-autonomously and can induce both responses in the surround (Figure 6C, 6D).

These results link reception of the FF signal by Ft and Ds, via D, the Wts-Hpo pathway, and Yki, to activation of the QE.

### D Is Required to Transduce the FF Signal

Of the various cytosolic components that function downstream of Ft and Ds, D is distinct in that it functions to promote, rather than to prevent, nuclear action of Yki and that it acts by repressing, rather than facilitating, Wts kinase activity [18],[19],[24],[49]. Hence, in the absence of D, Wts is constitutively active and Yki is excluded from the nucleus, irrespective of Ft-Ds signaling. Accordingly, removal of D should block transduction of the FF signal, preventing the recruitment of non-wing cells into the wing primordium. To test this, we performed the following four experiments.

First, we examined the consequences of generating *ds^o^ ap^o^*, *ft^o^ ap^o^*, and *ds^o^ ft^o^ ap^o^* discs that are also null for *d*. Discs of all three genotypes appear indistinguishable from *ap^o^* discs (unpublished data), as expected if D is not available to block Wts activity in the absence of Ds and/or Ft.

**Figure 7:**
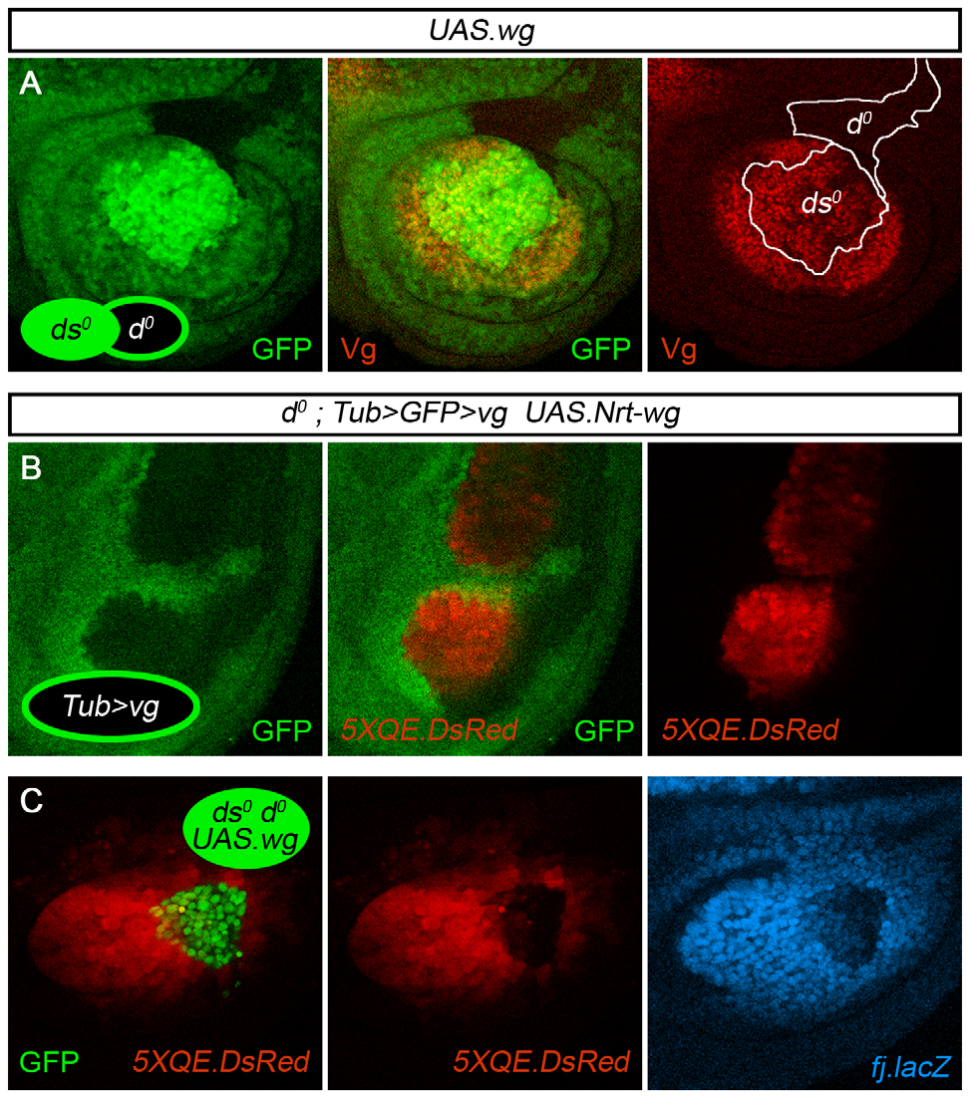
Dachs is required to receive, but not to send, feed-forward signal. (A) Sibling clones of *ds^o^* and *d^o^* cells in an *UAS.wg ap^o^* disc (clones marked by 2× and 0× GFP expression, respectively, as in Figure 5A, and outlined in white). The *ds^o^* clone expresses Vg and has induced Vg expression in abutting wild type cells but not in abutting *d^o^* cells. As a consequence, the latter are unable to contribute to the rescued wing primordium. This result contrasts with the behavior of wild type clones that are generated as siblings of *ds^o^* clones: as shown in Figure 4D, cells within such wild type clones can respond by activating the QE and joining the wing primordium. (B) Two clones of *Tub>vg* cells in an *UAS.Nrt-wg d^o^ ap^o^* disc. Both clones express the *5XQE-DsRed* reporter cell-autonomously and have induced a few adjacent cells to do the same, in marked contrast to the long-range propagation of QE-dependent Vg expression associated with *Tub>vg* clones generated in *UAS.Nrt-wg ap^o^* discs that retain wild type *d* function (Figures 1G, 2G). (C) A *UAS.wg ds^o^ d^o^* clone in an *ap^o^* disc. The clone has induced the long-range propagation of *5XQE-DsRed* and *fj-lacZ* expression in surrounding cells, but cells within the clone have failed to respond, or express only low levels of both reporters, indicating that they can send, but not receive, the FF signal.

Second, we generated twin spots of sibling *ds^o^* and *d^o^* clones in *UAS.wg ap^o^* discs. Under these conditions, the *ds^o^* clones both expressed Vg and induced Vg expression in neighboring wild type cells but failed to induce detectable expression in abutting cells belonging to the *d^o^* clone, resulting in their exclusion from the rescued wing pouch (Figure 7A).

Third, we generated clones of *Tub>vg* cells in both *ap^o^* and *d^o^ ap^o^* discs supplemented with uniform Nrt-Wg (as in Figure 1G). Such clones express peak levels of Vg and induce a long-range propagation of Vg and *5XQE.DsRed* expression in *ap^o^* discs (Figure 2G; [33]) but only a poorly penetrant and local induction of *5XQE.DsRed* expression in abutting cells in *d^o^ ap^o^* discs (Figure 7B).

Finally, we tested if the requirement for D in activating the QE is specific to transduction of the FF signal in “receiving” cells as opposed to production of the FF signal in “sending” cells by generating clones of *ds^o^ d^o^* double mutant clones that co-express *UAS.wg* in *ap^o^* discs. Such clones behave like corresponding clones of *ds^o^* single mutant cells (Figure 4D) in that they induce *5XQE.DsRed* expression in surrounding cells (Figure 7C). However, cells within the clone show either no or only low levels of *5XQE.DsRed* expression.

We conclude that the loss of D activity severely and selectively compromises the capacity of non-wing cells to transduce the FF signal, blocking activation of the QE and recruitment into the wing primordium.

## Discussion

**Figure 8:**
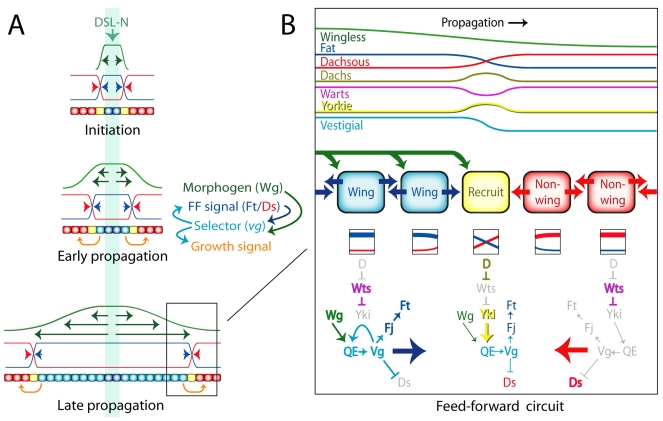
The *vestigial* feed-forward circuit, and the control of wing growth by morphogen. (A) A model for the control of wing growth by Wg. Initiation (top): the main phase of wing growth begins with segregation of the wing disc into D-V compartments and the induction of specialized border cells (dark blue) by DSL-Notch signaling (mint green): Notch activity drives expression of both the morphogen Wg (dark green) as well as Boundary enhancer (BE) dependent expression of the wing selector gene *vg*. As detailed in (B), Vg activity up-regulates Ft signaling (blue) at the expense of Ds signaling (red) to generate the feed-forward (FF) signal. The FF signal then acts together with Wg to induce Quadrant enhancer (QE) expression of *vg* in non-wing cells (red), initiating a stable circuit of Wg-dependent *vg* expression that recruits the responding non-wing cell (yellow) into the wing primordium. Early propagation (middle): Vg activity in newly recruited wing cells (turquoise) generates new FF signal, which acts together with Wg secreted by border cells to induce QE-dependent *vg* expression in neighboring non-wing cells. It also leads to the production of an additional “growth” signal (orange arrows) that promotes proliferation of the surrounding population of non-wing cells from which new wing cells will be recruited. As shown to the right, Vg activity and FF signaling comprise an auto-regulatory cycle driven by Wg. Each turn of the cycle corresponds to the recruitment of a non-wing cell into the wing primordium and generates a new, non-wing cell for subsequent recruitment. Late propagation (bottom): The wing primordium increases in size, propelled by propagation of the FF recruitment cycle and proliferation of cells within and around the primordium, both fueled by Wg as it spreads from D-V border cells. FF forward propagation also depends on Dpp spreading from border cells along the A-P compartment boundary, which acts together with Wg to promote the outward growth of the wing primordium from the intersection between the D-V and A-P compartment boundaries (not depicted; Figure 1A). (B) The feed-forward circuit. Top: the signaling activities of Wg, Ft, and Ds as well as the transducing activities of D, Wts, and Yki are shown relative to *vg* transcription, and the recruitment of non-wing cells into the wing primordium (recruitment propagates from left to right, coloring as in A). Away from the recruitment interface, Ft and Ds signaling activities are weakly graded or flat, D and Yki activities are low, and Wts activity is high. At the recruitment interface, Ft and Ds signaling activities are steeply graded and opposite, generating a transient pulse in D activity, a dip in Wts activity, and a burst of Yki activity. Middle: Wg, Ft, and Ds signals are shown as green, blue, and red arrows. Only the cell undergoing recruitment (yellow) receives both Wg as well as steep and opposing Ft and Ds signals. Bottom: the regulatory circuits underlying the wing (*vg* ^ON^; left) and non-wing (*vg* ^OFF^; right) states as well as the transition that occurs during recruitment (*vg* ^OFF^ to *vg* ^ON^; middle) are diagrammed relative to the landscapes of Wg, Ft, and Ds signaling upon which they depend. In wing cells, Wg input acts together with Vg to drive a positive auto-regulatory circuit of *vg* expression mediated by the Quadrant Enhancer (QE), and Vg up-regulates the expression of Fj while repressing that of Ds to enhance Ft signaling at the expense of Ds signaling (blue arrow). In non-wing cells, both the absence of Vg as well as the low level of nuclear Yki lead, by default, to low levels of Fj and high levels of Ds, enhancing Ds signaling at the expense of Ft signaling (red arrow). The box underneath each cell depicts the level and asymmetry of Ft and Ds inputs received from abutting cells on either side. Relatively uniform inputs (depicted by parallel lines in the boxes under wing and non-wing cells) cause modest, or no, polarization of the transducing activities of both proteins within each cell, suppressing the capacity of D to inhibit Wts activity and elevate nuclear import of Yki. Conversely, steep and opposite inputs (depicted by crossing lines in the box beneath the cell undergoing recruitment) cause a strong polarization, allowing D to inhibit Wts activity and induce a burst of Yki nuclear activity. Both Yki and Vg activate *vg* transcription via the QE by functioning as transcriptional co-activators for the same DNA binding protein, Sd (not depicted). Hence, as the level of Vg rises in cells undergoing recruitment, Vg can substitute for Yki to generate a stable circuit of Wg-dependent Vg auto-regulation that no longer requires Yki or FF input. Note that the depictions of *vg* expression, as well as of Ft and Ds signaling, as uniform away from the recruitment interface are simplifications. Instead, *vg* expression is weakly graded within the wing primordium in response to graded Wg signal, and the complementary patterns of *fj* and *ds*, upon which the signaling activities of Ft and Ds depend, are similarly graded. The resulting shallow differentials of Ft and Ds signaling may suffice to polarize cells in the plane of the epithelium (PCP). Nevertheless, the expression profiles of all three genes show a dramatic increase in steepness at the periphery of the wing primordium, and it is the resulting steepness in opposing Ds and Ft signals that we posit is essential to induce the burst of Yki nuclear activity upon which recruitment depends.

During larval life, the *Drosophila* wing primordium undergoes a dramatic ∼200-fold increase in cell number and mass driven by the morphogens Wg and Dpp. Focusing on Wg, we previously established that this increase depends at least in part on a reiterative process of recruitment in which wing cells send a FF signal that induces neighboring cells to join the primordium in response to morphogen [33],[34]. As summarized in Figure 8, our present results identify Ft-Ds signaling, the Wts-Hpo tumor suppressor pathway, and the transcriptional co-activator Yki as essential components of the FF process and define the circuitry by which it propagates from one cell to the next. We consider, in turn, the nature of the circuit, the parallels between FF signaling and PCP, and the implications for the control of organ growth by morphogen.

### The *vg* FF Circuit

#### Sending the FF signal

We present several lines of evidence that expression of the wing selector gene *vg* drives production of the FF signal by promoting a non-autonomous signaling activity of Ft. First, we show that *vg* acts both to up-regulate *fj* and down-regulate *ds*, two outputs known to elevate an outgoing, signaling activity of Ft in PCP [20],[21],[23]. Second, we demonstrate that experimental manipulations that elevate Ft signaling—specifically, over-expression of Ft or removal of Ds—generate ectopic FF signal. Third, and most incisively, we show that *ft* is normally essential in wing cells to send FF signal.

#### Receiving the FF signal

We show that Ft and Ds are both required in non-wing cells to receive the FF signal, functioning in this capacity to prevent the activation of *vg* unless countermanded by FF input. Notably, the removal of either Ft or Ds from non-wing cells constitutively activates the FF signal transduction pathway, mimicking receipt of the FF signal. However, the pathway is only weakly activated in this condition and the cells are refractory to any further elevation in pathway activity.

#### Transducing the FF signal

Previous studies have defined a transduction pathway that links Ft-Ds signaling via the atypical myosin D to suppression of the Wts kinase and enhanced nuclear import of Yki [9],[10],[11],[12],[18],[19],[49]. Likewise, we find that Ft and Ds operate through the same pathway to transduce the FF signal. Specifically, we show that manipulations of the pathway that increase nuclear activity of Yki (over-expression of D or Yki, or loss of Wts or Ex) cause non-wing cells to adopt the wing state. Conversely, removal of D, an intervention that precludes down-regulation of Wts by Ft-Ds signaling, prevents non-wing cells from being recruited into the wing primordium.

#### Recruitment

To induce non-wing cells to become wing cells, transduction of the FF signal has to activate *vg* transcription. Activation is mediated by the *vg* QE [33],[34],[35] and depends on binding sites for Scalloped (Sd), a member of the TEAD/TEF family of DNA binding proteins that can combine with either Yki or Vg to form a transcriptional activator [50],[51],[52],[53],[54],[55],[56],[57]. Hence, we posit that Yki transduces the FF signal by entering the nucleus and combining with Sd to activate *vg*. In addition, we posit that once sufficient Vg produced under Yki-Sd control accumulates, it can substitute for Yki to generate a stable auto-regulatory loop in which Vg, operating in complex with Sd, sustains its own expression. Accordingly, we view recruitment as a ratchet mechanism. Once the auto-regulatory loop is established, neither FF signaling nor the resulting elevation in Yki activity would be required to sustain *vg* expression and maintain the wing state (Figure 8).

#### Morphogen as fuel for FF propagation

Both the activation of the QE by Yki as well as the maintenance of its activity by Vg depend on Wg and Dpp input [33],[34],[35],[50] and hence define distinct circuits of *vg* auto-regulation fueled by morphogen. For activation, the circuit is inter-cellular, depending on Ft-Ds signaling for *vg* activity to propagate from one cell to the next. For maintenance, the circuit is intra-cellular, depending on Vg to sustain its own expression. Accordingly, we posit that growth of the wing primordium is propelled by the progressive expansion in the range of morphogen, which acts both to recruit and to retain cells in the primordium (as diagrammed for Wg in Figure 8).

### Ft-Ds Signaling: Parallels between FF Propagation and PCP

To date, Ft-Ds signaling has been studied in two contexts: the control of Yki target genes in tissue growth and the orientation of cell structures in PCP. Most work on tissue growth has focused on Yki target genes that control basic cell parameters, such as survival, mass increase, and proliferation (e.g., *diap*, *bantam*, and *cyclinE*). In this context, Ds and Ft are thought to function as a ligand-receptor pair, with tissue-wide gradients of Ds signal serving to activate Ft to appropriate levels within each cell [11],[18],[19],[24],[25]. In contrast, Ft and Ds behave as dual ligands and receptors in PCP, each protein having intrinsic and opposite signaling activity and both proteins being required to receive and orient cells in response to each signal [23],[32].

Here, we have analyzed a different, Yki-dependent aspect of growth, namely the control of organ size by the regulation of a selector gene, *vg*. In this case, Ft appears to correspond to a ligand, the FF signal, and Ds to a receptor required to receive the ligand—the opposite of the Ds-Ft ligand-receptor relationship inferred to regulate other Yki target genes. Moreover, as in PCP, we also find evidence that Ft and Ds operate as bidirectional ligands and receptors: like Ds, Ft is also required for receipt of the FF signal, possibly in response to an opposing signal conferred by Ds (Figure 8).

Studies of Ft-Ds interactions, both in vivo and in cell culture, have established that Ft and Ds interact in trans to form hetero-dimeric bridges between neighboring cells, the ratio of Ft to Ds presented on the surface of any given cell influencing the engagement of Ds and Ft on the abutting surfaces of its neighbors [28],[30],[44],[58]. These interactions are thought, in turn, to polarize the sub-cellular accumulation and activity of D [19],[24]. Accordingly, we posit that *vg* activity generates the FF signal by driving steep and opposing differentials of Ft and Ds signaling activity between wing (*vg* ^ON^) and non-wing (*vg* ^OFF^) cells. Further, we posit that these differentials are transduced in cells undergoing recruitment (yellow cells in Figure 8) by the resulting polarization of D activity, acting through the Wts-Hpo pathway and Yki to activate *vg*.

Thus, we propose that FF propagation and PCP depend on a common mechanism in which opposing Ft and Ds signals polarize D activity, both proteins acting as dual ligands and receptors for each other. However, the two processes differ in the downstream consequences of D polarization. For FF propagation, the degree of polarization governs a transcriptional response, via regulation of the Wts-Hpo pathway and Yki. For PCP, the direction of polarization controls an asymmetry in cell behavior, through a presently unknown molecular pathway.

FF propagation and PCP may also differ in their threshold responses to D polarization. We note that Figure 8 portrays *vg* expression and Ft-Ds signaling in an overly simplified form, in which the landscape is flat within frank wing and non-wing territories and steeply graded at the wing periphery, where recruitment occurs. In reality, *vg* expression is also graded, albeit weakly, within the wing primordium, due to the response of the QE to graded Wg and Dpp inputs [4],[50]. Hence, a shallow differential of Ft-Ds signaling reflecting that of Vg may be sufficient to orient cells in most of the prospective wing territories, but only cells in the vicinity of the recruitment interface may experience a steep enough differential to induce Yki to enter the nucleus and activate *vg*.

Finally, FF propagation and PCP differ in at least one other respect, namely, that they exhibit different dependent relationships between Ft and Ds signaling. In PCP, clonal removal of either Ft or Ds generates ectopic polarizing activity, apparently by creating an abrupt disparity in the balance of Ft-to-Ds signaling activity presented by mutant cells relative to that of their wild type neighbors [23]. By contrast, in FF propagation, only the removal of Ds, and not that of Ft, generates ectopic FF signal (Figure 4A–D). We attribute this difference to the underlying dependence of Ft and Ds signaling activity on *vg*. In *ds^o^* cells, Ft signaling activity is promoted both by the absence of Ds and by the Vg-dependent up-regulation of *fj*. However, in *ft^o^* cells, Ft is absent and Vg down-regulates *ds*, rendering the cells equivalent to *ds^o^ ft^o^* cells (which are devoid of signaling activity in PCP [23]). Thus, for FF propagation, the underlying circuitry creates a context in which only the loss of Ds, but not that of Ft, generates a strong, ectopic signal. For PCP, no such circuit bias applies.

### FF Signaling, the Steepness Hypothesis, and the Control of Growth by Morphogen

Morphogens organize gene expression and cell pattern by dictating distinct transcriptional responses at different threshold concentrations, a process that is understood conceptually, if not in molecular detail. At the same time, they also govern the rate at which developing tissues gain mass and proliferate, a process that continues to defy explanation.

One long-standing proposal, the “steepness hypothesis,” is that the slope of a morphogen gradient can be perceived locally as a difference in morphogen concentration across the diameter of each cell, providing a scalar value that dictates the rate of growth [26],[59],[60]. Indeed, in the context of the *Drosophila* wing, it has been proposed that the Dpp gradient directs opposing, tissue-wide gradients of *fj* and *ds* transcription, with the local differential of Ft-Ds signaling across every cell acting via D, the Wts-Hpo pathway, and Yki to control the rate of cell growth and proliferation [24],[25],[26]. The steepness hypothesis has been challenged, however, by experiments in which uniform distributions of morphogen, or uniform activation of their receptor systems, appear to cause extra, rather than reduced, organ growth [61],[62].

Our results provide an alternative interpretation. As discussed above and illustrated in Figure 8, we posit that “steepness,” as conferred by the local differential of Ft-Ds signaling across each cell, is not a direct reflection of morphogen slope but rather an indirect response governed by *vg* activity. Moreover, we propose that it promotes wing growth not by functioning as a relatively constant parameter to set a given level of Wts-Hpo pathway activity in all cells but rather by acting as a local, inductive cue to suppress Wts-Hpo pathway activity and recruit non-wing cells into the wing primordium.

How important is such local Ft-Ds signaling and FF propagation to the control of wing growth by morphogen? In the absence of D, cells are severely compromised for the capacity to transduce the FF signal (Figure 7), and the wing primordium gives rise to an adult appendage that is around a third the normal size, albeit normally patterned and proportioned [19]. A similar reduction in size is also observed when QE-dependent *vg* expression is obviated by other means [34]. Both findings indicate that FF signaling makes a significant contribution to the expansion of the wing primordium driven by Wg and Dpp. Nevertheless, wings formed in the absence of D are still larger than wings formed when either Wg or Dpp signaling is compromised [4],[5],[6],[7]. Hence, both morphogens must operate through additional mechanisms to promote wing growth.

Previously, we identified at least three other outputs of signaling by Wg (and likely Dpp) that work in conjunction with FF propagation [33],[34]. First, as discussed above, Wg is required to maintain *vg* expression in wing cells once they are recruited by FF signaling, and hence to retain them within the wing primordium. Second, it functions to provide a tonic signal necessary for wing cells to survive, gain mass, and proliferate at a characteristic rate (see also [62]). And third, it acts indirectly, via the capacity of wing cells, to stimulate the growth and proliferation of neighboring non-wing cells, the source population from which new wing cells will be recruited. All of these outputs, as well as FF propagation, depend on, and are fueled by, the outward spread of Wg and Dpp from D-V and A-P border cells. Accordingly, as we argue above, we think that wing growth is governed by the progressive expansion in the range of Wg and Dpp signaling.

### Cell Fate Specification, Wts-Hpo Pathway Activity, and the Control of Organ Size

Our identification of Ft-Ds signaling, the Wts-Hpo pathway, and Yki as key components of the FF recruitment process provides a striking parallel with the recently discovered involvement of the Wts-Hpo pathway and Yki/YAP in regulating primordial cell populations in vertebrates, notably the segregation of trophectoderm and inner cell mass in early mammalian embryos [63] and that of neural and endodermal progenitor cells into spinal cord neurons and gut [57],[64]. As in the *Drosophila* wing, Wts-Hpo activity and YAP appear to function in these contexts in a manner that is distinct from their generic roles in the regulation of cell survival, growth, and proliferation, namely as part of an intercellular signaling mechanism that specifies cell type. We suggest that this novel employment of the pathway constitutes a new, and potentially general, mechanism for regulating tissue and organ size.

## Materials and Methods

### Generation and Analysis of Mutant Clones

(i) Flp/FRT mediated mitotic recombination [65],[66], (ii) “flp-out cassette” excision [67],[68],[69], and (iii) Mosaic analysis with a repressible cell marker (MARCM [48]) techniques were used, in conjunction with the Gal4/UAS method [70], to manipulate gene function in genetically marked clones of cells in developing wing imaginal discs (e.g., as in [33],[34]).

Animals were cultured at 25°C, and clones were induced during the first larval instar (24–48 h after egg laying) by heat shock induced expression of an *Hsp70.flp* transgene (usually 36°C for 20 min). Wing discs from mature third instar larvae were dissected, fixed, and processed for immuno-fluorescence by standard methods, using anti-Vg, anti-Wg, anti-HA, and anti-βGal antisera (as in Zecca and Struhl, 2007a,b [33],[34]).

*vg* QE activity was monitored by expression of *1XQE.lacZ* and *5XQE.DSRed* reporter transgenes as well as by the expression of Vg protein in the absence of DSL-Notch signaling [33],[34],[35]. In some experiments, expression of the *fj-lacZ* enhancer trap allele *fj^P1^* [71], which is strongly up-regulated under Vg control, was also used in the absence of DSL-Notch input as a proxy for QE-dependent *vg* expression. All four assays gave similar results, with the *5XQE.DSRed* and *fj-lacZ* reporters showing the greatest sensitivity.

The following amorphic mutant alleles and transgenes were employed (http://flybase.bio.indiana.edu/) [9],[19],[22],[24],[28],[33],[34]:

Mutant alleles: ap^56f^, d^GC13^, *Df(2L)Exel6006*, *ds^UA071^*, *ds^2D60b^*, *ex^E1^*, *fj^P1^*, *ft^15^*, *fz^P21^*, *Dfz2 ^C1^*, *vg^83b27R^*, and *wts^X1^*.

Transgenes: *UAS.N^intra^*, *UAS.Nrt-wg*, *UAS.wg*, *Tubα1>GFP*,*y+>vg*, *C765.Gal4*, *nub.Gal4*, *Tubα1>Gal80>Gal4*, *UAS.ds^GS^*, *UAS.ft*, *UAS.d*, *UAS.yki*, *Hsp70.GFP*.

Exact genotypes, by Figure panel:

(2A) *y w 5XQE.DsRed/y w Hsp70.flp; FRT39 ap^56f^ fj^P1^/+.*

(2B) *y w 5XQE.DsRed/y w Hsp70.flp; FRT39 ap^56f^ fj^P1^/FRT39 ap^56f^.*

(2C) *y w 5XQE.DsRed/y w Hsp70.flp; ds^2D60b^ FRT39 ap^56f^ vg^83b27R^/+.*

(2D) *y w 5XQE.DsRed/y w Hsp70.flp; ds^2D60b^ FRT39 ap^56f^ vg^83b27R^/FRT39 ap^56f^.*

(2E) *y w Hsp70.flp/y w Hsp70.flp; ap^56f^ vg^83b27R^ 5XQE.DsRed/FRT39 ap^56f^ vg^83b27R^ fj^P1^; Tubα1>flu-GFP,y^+^>vg/+.*

(2F) *y w Hsp70.flp/y w Hsp70.flp; ap^56f^ vg^83b27R^ 5XQE.DsRed/ds^2D60b^ FRT39 ap^56f^ vg^83b27R^; Tubα1>flu-GFP,y^+^>vg/+.*

(2G) *y w 5XQE.DsRed/y w Hsp70.flp; FRT39 ap^56f^/Hsp70.flu-GFP FRT39 ap^56f^ fj^P1^; Tubα1>flu-GFP,y^+^>vg UAS.Nrt-flu-wg/C765.Gal4.*

(2H) *y w 5XQE.DsRed/y w Hsp70.flp; FRT39 ap^56f^/ds^2D60b^ FRT39 ap^56f^ vg^83b27R^; Tubα1>flu-GFP,y^+^>vg UAS.Nrt-flu-wg/C765.Gal4.*

(3A) *y w 5XQE.DsRed/y w Hsp70.flp; ds^UA071^ FRT39 ap^56f^/ds^UA071^ FRT39 ap^56f^; UAS.wg/+.*

(3B) *y w 5XQE.DsRed/y w 5XQE.DsRed; ft^15^ FRT39 ap^56f^/ds^UA071^ ft^15^ FRT39 ap^56f^ fj^P1^.*

(3C) *y w 5XQE.DsRed/y w 5XQE.DsRed; ds^UA071^ ft^15^ FRT39 ap^56f^/ds^UA071^ ft^15^ FRT39 ap^56f^ fj^P1^; Tubα1>CD2,y^+^>Gal4/+.*

(3D) *y w 5XQE.DsRed/y w Hsp70.flp; ft^15^ FRT39 ap^56f^/ft^15^ FRT39 ap^56f^; fz^P21^ Dfz2^C1^ FRT2A/Hsp70.CD2 Hsp70.flu-GFP FRT2A.*

(3E) *y w 5XQE.DsRed/y w Hsp70.flp; ft^15^ FRT39 ap^56f^/ft^15^ FRT39 ap^56f^; UAS>CD2,y^+^>Nrt-flu-wg C765.Gal4/+.*

(4A) *y w 5XQE.DsRed/y w Hsp70.flp Tuba1.Gal4 UAS.GFPnls; ds^UA071^ FRT39 ap^56f^/Hsp70.flu-GFP Tubα1.Gal80 FRT39 ap^56f^ fj^P1^.*

(4B) *y w 5XQE.DsRed/y w Hsp70.flp; ft^15^ FRT39 ap^56f^/Hsp70.flu-GFP Tubα1.Gal80 FRT39 ap^56f^ fj^P1^; UAS.wg/+.*

(4C) *y w 5XQE.DsRed/y w Hsp70.flp; ds^UA071^ ft^15^ FRT39 ap^56f^ fj^P1^/Hsp70.flu-GFP Tubα1.Gal80 FRT39 ap^56f^; C765.Gal4/+.*

(4D) *y w 5XQE.DsRed/y w Hsp70.flp; ds^UA071^ FRT39 ap^56f^/Hsp70.flu-GFP FRT39 ap^56f^; UAS.Nrt-flu-wg/C765.Gal4.*

(4E) *y w 5XQE.DsRed/y w Hsp70.flp; ft^15^ FRT39 ap^56f^/Hsp70.flu-GFP FRT39 ap^56f^; UAS.Nrt-flu-wg/C765.Gal4.*

(4F) *y w Hsp70.flp/y w Hsp70.flp; ds^UA071^ FRT39 ap^56f^/Hsp70.flu-GFP FRT39 ap^56f^; UAS>CD2,y^+^>Nrt-flu-wg C765.Gal4/1XQE.lacZ.*

(5A) *y w 5XQE.DsRed/y w Hsp70.flp; ds^UA071^ Tubα1.Gal80 FRT39 ap^56f^ vg^83b27R^/Hsp70.flu-GFP FRT39 ap^56f^ fj^P1^; UAS.ft/Tuba1.Gal4.*

(5B) *y w 5XQE.DsRed/y w Hsp70.flp; ft^15^ Tubα1.Gal80 FRT39 ap^56f^/Hsp70.flu-GFP FRT39 ap^56f^ fj^P1^; UAS.ft/Tubα1.Gal4.*

(5C) *y w 5XQE.DsRed/y w Hsp70.flp UAS.GFPnls; FRT39 ap^56f^ fj^P1^/FRT39 ap^56f^ UAS.flu-wg; UAS.ft/Tubα1>Gal80,y^+^>Gal4.*

(5D) *y w 5XQE.DsRed/y w Hsp70.flp UAS.GFPnls; ap^56f^ 1XQE.lacZ/ds^UA071^ FRT39 ap^56f^; UAS.ds/Tubα1>Gal80,y^+^>Gal4 UAS.wg.*

(6A) *y w 5XQE.DsRed/y w Hsp70.flp Tuba1.Gal4 UAS.GFPnls; FRT39 ap^56f^/Hsp70.flu-GFP Tubα1.Gal80 FRT39 ap^56f^ fj^P1^; UAS.d/UAS.wg.*

(6B) *y w 5XQE.DsRed/y w Hsp70.flp UAS.GFPnls; FRT39 ap^56f^ UAS.flu-wg/FRT39 ap^56f^ fj^P1^; Tubα1>Gal80,y^+^>Gal4 UAS.yki/+.*

(6C) *y w Hsp70.flp/y w Hsp70.flp; nub-Gal4 FRT39 ap^56f^/ap^56f^ UAS.flu-Nrt-wg; FRT82 wts^x1^/FRT82 Hsp70.flu-GFP.*

(6D) *y w 5XQE.DsRed/y w Hsp70.flp; ex^e1^ FRT39 ap^56f^/Hsp70.flu-GFP FRT39 ap^56f^ fj^P1^; UAS.wg/C765.Gal4.*

(7A) *y w 5XQE.DsRed/y w Hsp70.flp; ds^UA071^ Hsp70.flu-GFP FRT39 ap^56f^/d^GC13^ FRT39 ap^56f^ fj^P1^; UAS.wg/C765.Gal4.*

(7B) *y w 5XQE.DsRed/y w Hsp70.flp; d^GC13^ FRT39 ap^56f^ fj^P1^/d^GC13^ FRT39 ap^56f^; Tubα1>flu-GFP,y^+^>vg UAS.Nrt-flu-wg/C765.Gal4.*

(7C) *y w 5XQE.DsRed/y w Hsp70.flp Tuba1.Gal4 UAS.GFPnls; ds^UA071^ d^GC13^ FRT39 ap^56f^/Hsp70.flu-GFP Tuba1.Gal80 FRT39 ap^56f^ fj^P1^; UAS.wg/+.*

(S1A) *y w 5XQE-DsRed/y w Hsp70.flp; ds^UA071^ FRT39 ap^56f^/ds^UA071^ FRT39 ap^56f^; UAS.Nrt-flu-wg/Tubα1>Gal80,y^+^>Gal4.*

(S1B) *y w Hsp70.flp/y w Hsp70.flp; ap^56f^ UAS>CD2,y^+^>Nrt-flu-wg/nub-Gal4 FRT39 ap^56f^; FRT82 wts^x1^/FRT82 Hsp70.flu-GFP.*

(S1C) *y w Hsp70.flp/y w Hsp70.flp; ap^56f^ 1XQE.lacZ/FRT39 ap^56f^; UAS>CD2,y^+^>Nrt-flu-wg C765.Gal4/UAS.yki.*

(S2A) as (4E).

(S2B) *y w 5XQE-DsRed/y w Hsp70.flp; ft^15^ FRT39 ap^56f^/Df(2L)Exel6006 Hsp70.flu-GFP FRT39 ap^56f^; UAS.Nrt-flu-wg/C765.Gal4.*

(S2C) *y w Hsp70.flp/y w Hsp70.flp; ft^15^ Tuba1.Gal80 FRT39 ap^56f^/Hsp70.flu-GFP FRT39 ap^56f^; UAS>CD2,y^+^>Nrt-flu-wg C765.Gal4/1XQE.lacZ.*

(S3A) *y w Hsp70.flp Tubα1.Gal4 UAS-GFPnls/y w Hsp70.flp; wg^cx4^ FRT39 ap^56f^/Hsp70.flu-GFP Tubα1.Gal80 FRT39 ap^56f^; UAS.N^intra^/1XQE.lacZ.*

(S3B) *y w 5XQE-DsRed/y w Hsp70.flp Tubα1.Gal4 UAS-GFPnls; ft^15^ wg^cx4^ FRT39 ap^56f^/Hsp70.flu-GFP Tubα1.Gal80 FRT39 ap^56f^; lqf^1227^ Hsp70-CD2 FRT2A UAS.N^intra^/+.*

(S3C) *y w Hsp70.flp Tubα1.Gal4 UAS-GFPnls/y w Hsp70.flp; ft^15^ FRT39 ap^56f^/Tubα1.Gal80 FRT39; UAS.wg/+.*

(S3D) *y w omb-lacZ/y w Hsp70.flp Tubα1.Gal4 UAS-GFPnls; ft^15^ FRT39 ap^56f^/Tubα1.Gal80 FRT39; UAS.dpp/+.*

## Supporting Information

Figure S1 **Quadrant enhancer activity is Wingless dependent in** ***ap^o^*** **discs that lack either Dachsous or Warts or that over-express Yorkie.** (A) A *UAS.Nrt-wg* clone in a *ds^o^ ap^o^* disc. Both Vg and *5XQE.DsRed* are expressed at peak levels in the clone and in surrounding cells that abut the clone, as observed for UAS.Nrt-wg clones in *ft^o^ ap^o^* discs (Figure 3E). (B) *UAS.Nrt-wg* clones generated in an *ap^o^* disc largely composed of *wts^o^* clonal tissue; as in (A), Vg is strongly up-regulated in the *UAS.Nrt-wg* clones and abutting cells. (C) *UAS.Nrt-wg* clones generated in an *UAS.yki ap^o^* disc; same outcome as in (A), except a *1XQE.lacZ* transgene was used instead of the *5XQE.DsRed* transgene.
(3.99 MB TIF)

Figure S2 **Exceptional cases of local non-autonomous Quadrant enhancer activity associated with** ***ft^o^*** **clones can be attributed to induction by their sibling 2×** ***ft^+^*** **clones.** (A) A *ft^o^* clone (marked by the absence of GFP) associated with local, non-autonomous activity of the *5XQE.DsRed* transgene (appears yellow in A') in an *ap^o^ UAS.Nrt-wg* disc. Note that this non-autonomous expression is associated with a sibling 2×*ft^+^* clone (white arrow, marked by 2× GFP expression in a 1× GFP 1×*ft^+^* background). In this experiment, 26/43 *ft^o^* clones were associated with strictly cell-autonomous QE activity (as in Figure 4E): of these, 12/26 had an associated 2×*ft^+^* twin (Figure 4E), and the remaining 14/26 clones had either no detectable twin (7/14) or a very small twin (<8 cells; 7/14). The remaining 17/43 *ft^o^* clones showed local QE activity in neighboring cells: in 7/17 cases, this non-autonomous activity was associated with a 2×*ft^+^* twin clone (as shown in this panel), and in the remaining 10/17 cases, 9/10 had no detectable twin, and 1/10 had a twin clone located elsewhere. Thus, the majority of *ft^o^* clones analyzed in this experiment showed a strictly cell-autonomous response, and in 7/8 cases in which local, non-autonomous *5XQE.DsRed* expression was observed and a 2×*ft^+^* twin survived, the twin spot was associated with the *5XQE.DsRed* expression. Based on these results, we attribute the exceptional cases of non-autonomous *5XQE.DsRed* expression associated with *ft^o^* clones to signaling by their 2×*ft^+^* sibling clones, a conclusion further supported by experiments in panels (B) and (C). (B) A *ft^o^* clone generated and marked as in (A), except under conditions in which its sibling 2×*ft^+^* clone died, owing to homozygosity for *Df(2L)Exel6006*. Note the strictly cell-autonomous expression of the *5XQE.DsRed* transgene. 39/45 clones generated in this experiment behaved in this way; 6/45 showed local non-autonomy. We have not determined how quickly the sibling 2×*ft^+^ Df(2L)Exel6006* clones die after being generated in this experiment; it is possible that rare 2×*ft^+^ Df(2L)Exel6006* clones survive long enough to induce self-sustaining *vg* and *5XQE.DsRed* expression in neighboring cells prior to their loss. (C) A *UAS.Nrt-wg 2*×*ft^+^* clone (marked by 2× GFP in a 1× GFP 1×*ft^+^* background, and outlined in white in the right panel) and its *ft^o^* sibling clone (marked by the absence of GFP) in an *ap^o^* disc. Note the association of the *2*×*ft^+^* clone with ectopic Vg expression as well as the local induction of Vg expression by Nrt-Wg in neighboring cells (Nrt-Wg expression was also assayed, independently, in this clone; unpublished data). This result corroborates the evidence shown in (A) and (B), that 2×*ft^+^* clones generated in 1×*ft^+^ ap^o^* discs have the capacity to induce *5XQE-DsRed* and *vg* expression.
(2.19 MB TIF)

Figure S3 ***ft^o^*** **clones can send Delta/Serrate/Lag2 (DSL), Wingless, and Decapentaplegic signals.** (A) A *UAS.N^intra^ wg^o^* clone generated in an *ap^o^* disc. *N^intra^* encodes a constitutively active form of Notch; clones of *UAS.N^intra^ wg^o^* cells in *ap^o^* discs up-regulate the expression of the Notch ligands Delta and Serrate and activate Notch in adjacent cells, as visualized by the induction of a ring of ectopic, Wg-expressing D-V border cells encircling the clone (no Wg is made within the clone, as it is *wg^o^*). These ectopic border cells suffice to initiate the long-range propagation of QE-dependent *vg* expression in surrounding cells, as indicated by the broad halo of *1XQE-lacZ* expression. (B) A *UAS.N^intra^ wg^o^ ft^o^* clone generated an *ap^o^* disc. Essentially the same experiment shown in (A), except that the clones are also *ft^o^*. The result is the same (except that a *5XQE-DsRed* reporter was used in place of the *1XQE-lacZ* reporter), indicating that cells in the clone can send DSL signals to the surround, even though they are devoid of Ft. (C) A *UAS.wg ft^o^* clone generated in a wild type disc. QE-dependent *vg* expression depends on the level of Wg input. As a consequence *UAS.wg* clones up-regulate Vg expression in surrounding cells within the wing pouch, as seen in this example, even though the clone is also *ft^o^*. (D) A *UAS.dpp ft^o^* clone generated in a wild type disc. Ectopic Dpp expressed by the clone has induced ectopic *omb-lacZ* expression in the surround, even though the clone is *ft^o^*.
(3.03 MB TIF)

## Abbreviations

*ap*: *apterous*
BE: Boundary Enhancer
BMP: Bone Morphogenetic Protein
D: Dachs
D: dorsal
Dpp: Decapentaplegic
Ds: Dachsous
Ex: Expanded
FF: feed-forward
*fj*: *four-jointed*
Ft: Fat
Hh: Hedgehog
Hpo: Hippo
Nrt: Neuroactin
PCP: planar cell polarity
QE: Quadrant Enhancer
Sav: Salvador
Sd: Scalloped
Tub: Tubulinα1
V: ventral
*vg*: *vestigial*
Wnt: Wingless/Int
Wts: Warts
YAP: YES Associated Protein
Yki: Yorkie
