# Supplementary figures and images for: A Feed-Forward Circuit Linking Wingless, Fat-Dachsous Signaling, and the Warts-Hippo Pathway to Drosophila Wing Growth
Source: PLoS Biol. 2010 Jun 1;8(6):e1000386. doi: 10.1371/journal.pbio.1000386 (PMC2879410; doi:10.1371/journal.pbio.1000386)

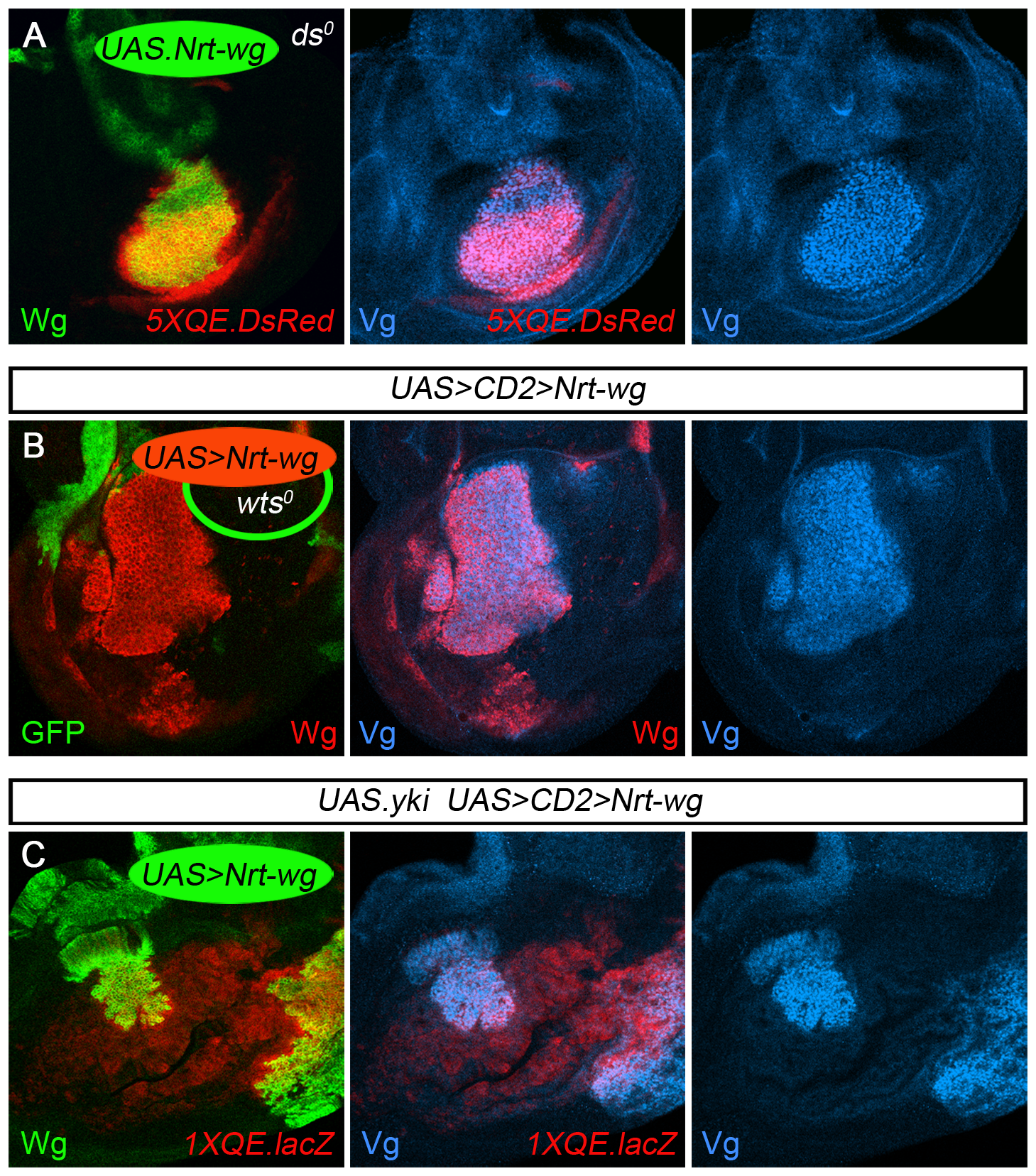

Supplement: Figure S1 — Quadrant enhancer activity is Wingless dependent in apo discs that lack either Dachsous or Warts or that over-express Yorkie. (A) A UAS.Nrt-wg clone in a dso apo disc. Both Vg and 5XQE.DsRed are expressed at peak levels in the clone and in surrounding cells that abut the clone, as observed for UAS.Nrt-wg clones in fto apo discs (Figure 3E). (B) UAS.Nrt-wg clones generated in an apo disc largely composed of wtso clonal tissue; as in (A), Vg is strongly up-regulated in the UAS.Nrt-wg clones and abutting cells. (C) UAS.Nrt-wg clones generated in an UAS.yki apo disc; same outcome as in (A), except a 1XQE.lacZ transgene was used instead of the 5XQE.DsRed transgene. (3.99 MB TIF) [file pbio.1000386.s001.tif]

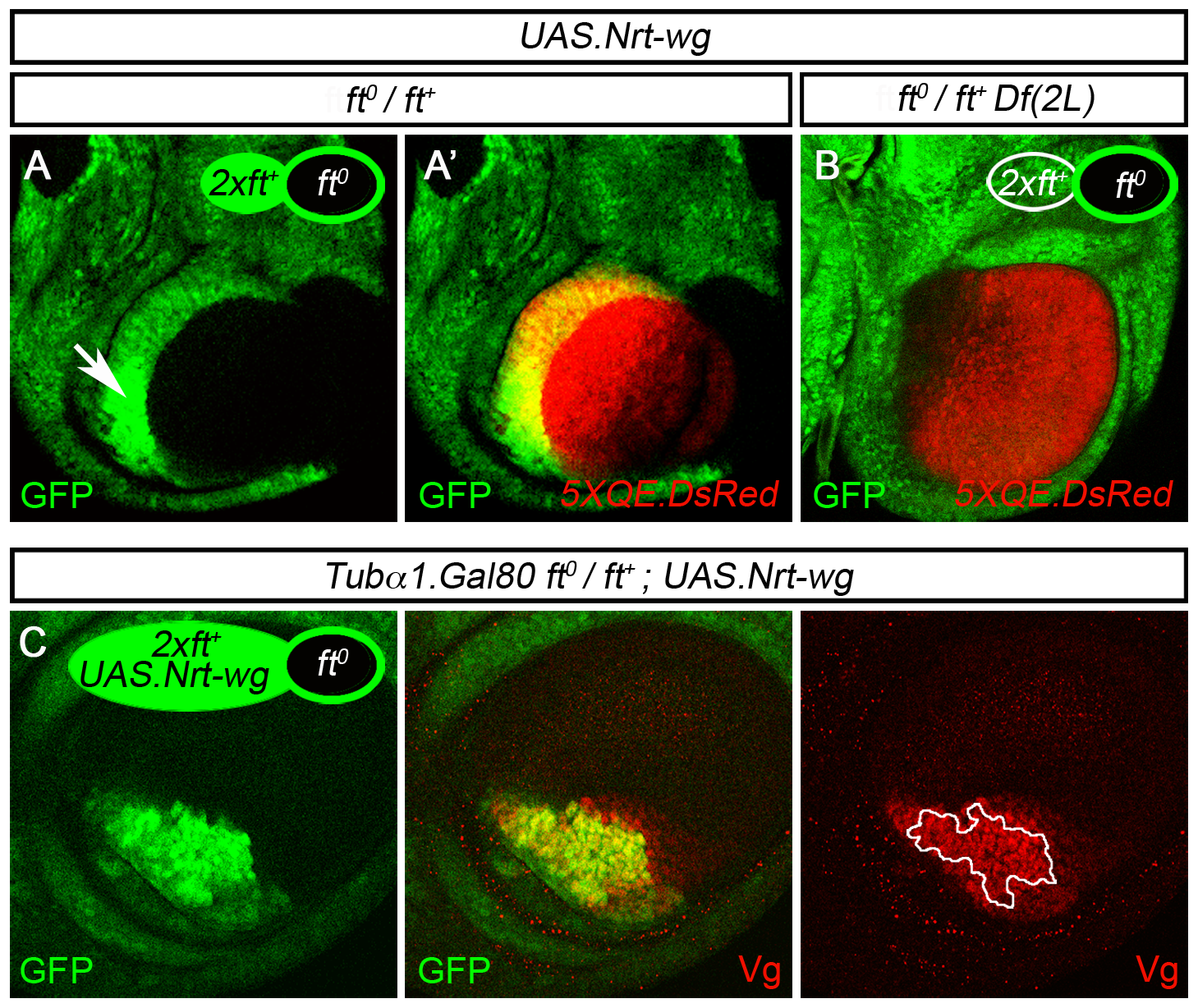

Supplement: Figure S2 — Exceptional cases of local non-autonomous Quadrant enhancer activity associated with fto clones can be attributed to induction by their sibling 2× ft+ clones. (A) A fto clone (marked by the absence of GFP) associated with local, non-autonomous activity of the 5XQE.DsRed transgene (appears yellow in A') in an apo UAS.Nrt-wg disc. Note that this non-autonomous expression is associated with a sibling 2×ft+ clone (white arrow, marked by 2× GFP expression in a 1× GFP 1×ft+ background). In this experiment, 26/43 fto clones were associated with strictly cell-autonomous QE activity (as in Figure 4E): of these, 12/26 had an associated 2×ft+ twin (Figure 4E), and the remaining 14/26 clones had either no detectable twin (7/14) or a very small twin (<8 cells; 7/14). The remaining 17/43 fto clones showed local QE activity in neighboring cells: in 7/17 cases, this non-autonomous activity was associated with a 2×ft+ twin clone (as shown in this panel), and in the remaining 10/17 cases, 9/10 had no detectable twin, and 1/10 had a twin clone located elsewhere. Thus, the majority of fto clones analyzed in this experiment showed a strictly cell-autonomous response, and in 7/8 cases in which local, non-autonomous 5XQE.DsRed expression was observed and a 2×ft+ twin survived, the twin spot was associated with the 5XQE.DsRed expression. Based on these results, we attribute the exceptional cases of non-autonomous 5XQE.DsRed expression associated with fto clones to signaling by their 2×ft+ sibling clones, a conclusion further supported by experiments in panels (B) and (C). (B) A fto clone generated and marked as in (A), except under conditions in which its sibling 2×ft+ clone died, owing to homozygosity for Df(2L)Exel6006. Note the strictly cell-autonomous expression of the 5XQE.DsRed transgene. 39/45 clones generated in this experiment behaved in this way; 6/45 showed local non-autonomy. We have not determined how quickly the sibling 2×ft+ Df(2L)Exel6006 clones die after being generated in [file pbio.1000386.s002.tif]

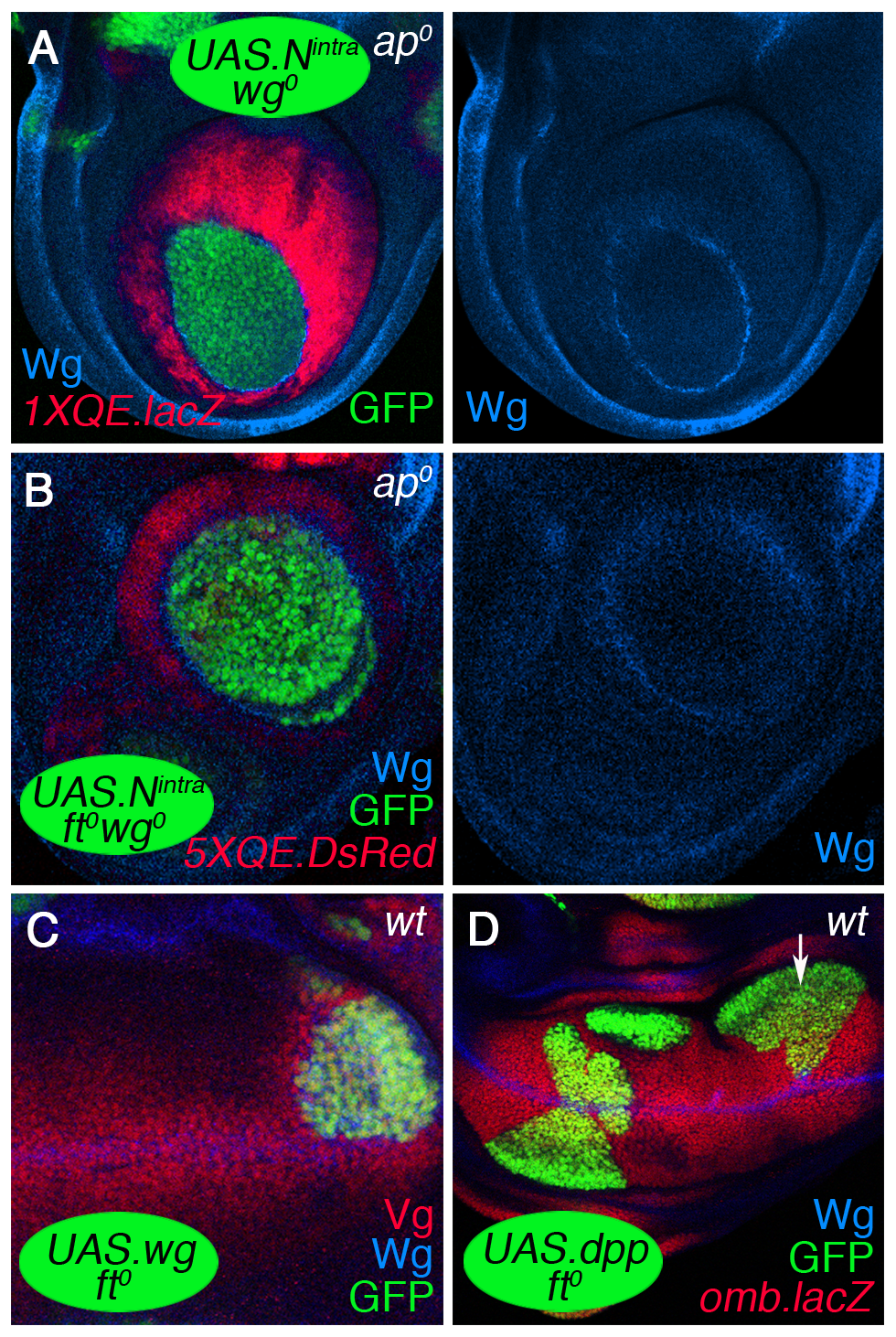

Supplement: Figure S3 — fto clones can send Delta/Serrate/Lag2 (DSL), Wingless, and Decapentaplegic signals. (A) A UAS.Nintra wgo clone generated in an apo disc. Nintra encodes a constitutively active form of Notch; clones of UAS.Nintra wgo cells in apo discs up-regulate the expression of the Notch ligands Delta and Serrate and activate Notch in adjacent cells, as visualized by the induction of a ring of ectopic, Wg-expressing D-V border cells encircling the clone (no Wg is made within the clone, as it is wgo). These ectopic border cells suffice to initiate the long-range propagation of QE-dependent vg expression in surrounding cells, as indicated by the broad halo of 1XQE-lacZ expression. (B) A UAS.Nintra wgo fto clone generated an apo disc. Essentially the same experiment shown in (A), except that the clones are also fto. The result is the same (except that a 5XQE-DsRed reporter was used in place of the 1XQE-lacZ reporter), indicating that cells in the clone can send DSL signals to the surround, even though they are devoid of Ft. (C) A UAS.wg fto clone generated in a wild type disc. QE-dependent vg expression depends on the level of Wg input. As a consequence UAS.wg clones up-regulate Vg expression in surrounding cells within the wing pouch, as seen in this example, even though the clone is also fto. (D) A UAS.dpp fto clone generated in a wild type disc. Ectopic Dpp expressed by the clone has induced ectopic omb-lacZ expression in the surround, even though the clone is fto. (3.03 MB TIF) [file pbio.1000386.s003.tif]
